# Artificial cells drive neural differentiation

**DOI:** 10.1126/sciadv.abb4920

**Published:** 2020-09-18

**Authors:** Ö. Duhan Toparlak, Jacopo Zasso, Simone Bridi, Mauro Dalla Serra, Paolo Macchi, Luciano Conti, Marie-Laure Baudet, Sheref S. Mansy

**Affiliations:** 1 Department CIBIO, University of Trento, via Sommarive 9, 38123 Povo, Italy.; 2 National Research Council—Institute of Biophysics & Bruno Kessler Foundation, via alla Cascata 56/C, 38123 Trento, Italy.; 3 Department of Chemistry, University of Alberta, 11227 Saskatchewan Drive, Edmonton, AB T6G 2G2, Canada.

## Abstract

We report the construction of artificial cells that chemically communicate with mammalian cells under physiological conditions. The artificial cells respond to the presence of a small molecule in the environment by synthesizing and releasing a potent protein signal, brain-derived neurotrophic factor. Genetically controlled artificial cells communicate with engineered human embryonic kidney cells and murine neural stem cells. The data suggest that artificial cells are a versatile chassis for the in situ synthesis and on-demand release of chemical signals that elicit desired phenotypic changes of eukaryotic cells, including neuronal differentiation. In the future, artificial cells could be engineered to go beyond the capabilities of typical smart drug delivery vehicles by synthesizing and delivering specific therapeutic molecules tailored to distinct physiological conditions.

## Teaser

Stimuli-responsive artificial cells synthesize and controllably release therapeutics for neural differentiation.

## INTRODUCTION

The tendency to view cells as autonomous units misses the fact that life depends on and interacts with other living organisms. This interaction can be remote and indirect, as those that govern the nitrogen cycle, or the interactions can be much more direct, as seen during fertilization. Nevertheless, most attempts at building cellular mimics from component parts, i.e., artificial cells, focus on reconstituting biological-like activity under laboratory conditions in the absence of other living cells (*1*, *2*). Conversely, a few laboratories have begun to assemble more complex ecosystems consisting of distinct artificial cells or artificial cells mixed with natural living cells (*3*–*10*). Artificial cells that integrate within the wider cellular community may help to uncover the physical-chemical underpinnings of cellular behavior and provide for opportunities to engineer advanced drug delivery systems (*11*, *12*). Despite the potential and future implications of this technology, artificial cells that interact with eukaryotic cells under physiological conditions have not been demonstrated thus far.

Here, we describe a new class of genetically controlled, stimuli-responsive artificial cells that chemically communicate with neurons and promote the differentiation of neural stem cells. The artificial cells are further capable of sending chemical messages to engineered human embryonic kidney (HEK) 293T cells. The artificial cells are specifically formulated to be functional under physiological conditions so as to facilitate future efforts in building medicinally useful artificial cells. The data suggest that artificial cells are a versatile chassis for the in situ synthesis and on-demand release of chemical signals. Artificial cells could be engineered to go beyond the capabilities of typical smart drug delivery vehicles by synthesizing and delivering specific therapeutic molecules tailored to local physiological conditions.

## RESULTS

### An artificial cellular chassis designed to integrate with eukaryotic cells

**Fig. 1:**
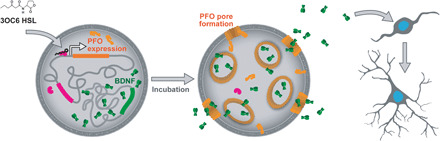
Communication between artificial and neural stem cells. The synthesis of PFO monomers (light orange) is controlled by a genetic AND-gate that requires both LuxR and 3OC6 HSL for gene expression. Monomers of PFO assemble into pores in the presence of cholesterol-containing membranes, thereby releasing BDNF. Homodimers of mature BDNF (green) act on the cognate receptor TrkB, activating signaling pathways leading to neural stem cell differentiation and maturation. The figure was not drawn to scale.

The artificial cells were housed within a phospholipid vesicle that mainly consisted of 1-palmitoyl-2-oleoyl-*sn*-glycero-3-phosphocholine (POPC) and cholesterol. The artificial cells contained transcription-translation machinery and DNA templates that coded for brain-derived neurotrophic factor (BDNF), LuxR, and perfringolysin O (PFO). BDNF is a neurotrophic factor that is critical to the development and function of the nervous system by promoting the survival and differentiation of neurons, leading to neurite outgrowth, branching, and synapse formation and stabilization (*13*–*16*). Intravesicularly synthesized BDNF escaped from the vesicle through protein pores. The monomeric subunits of PFO were expressed in soluble form inside the artificial cells and assembled into oligomeric pores in the presence of cholesterol-containing lipid membranes. The outer diameters of the BDNF homodimer and the PFO pore are 4 nm and 25 to 30 nm, respectively (*17*, *18*). Therefore, functional PFO pores allowed the escape of BDNF from the artificial cell. The expression of PFO was regulated by the *N*-3-oxohexanoyl homoserine lactone (3OC6 HSL)–responsive transcriptional repressor LuxR. In this arrangement, BDNF was always synthesized but only released from the artificial cell in the presence of 3OC6 HSL (Fig. 1).

### A physiologically compatible cell-free expression platform

A new in vitro formulation of transcription-translation machinery was developed and used for all of the described experiments. The toxicity of different expression systems was screened with a highly sensitive ex vivo collapse assay from the outset. This physiologically relevant assay measured the extent of toxicity-induced collapse of the leading tip of axons, i.e., the growth cone (fig. S1A). HeLa cell extract, rabbit reticulocyte lysate, and the PURE system (*19*) all increased growth cone collapse by more than twofold (fig. S1A). Although the *Escherichia coli* S12 crude cell extract gave data similar to that of phosphate-buffered saline (PBS) (fig. S1A), the osmolality of the fully assembled reaction (*20*) was too high (ca. 500 mOsm/kg H_2_O) (fig. S1C). For comparison, the osmolality of human plasma is 285 to 305 mOsm/kg H_2_O. Therefore, the composition of this nontoxic *E. coli* S12 extract was optimized so as to maintain the integrity of the artificial cells under physiological conditions (figs. S1 to S3).

The homemade and optimized S12 cell-free system was used for every experiment except for this initial screening of toxicity with the axonal collapse assay and the screening of the S12 reaction conditions (figs. S3, A and C, and S4, B, D, and E). After an initial assessment of reaction conditions monitored by the expression of green fluorescent protein (GFP) (figs. S1C and S2, A to E), the transcriptional promoters and template DNA concentrations were optimized to produce a maximal amount of BDNF and minimal amount of LuxR and PFO with the limited resources available within the artificial cell (fig. S2, F and G). Therefore, strong and weak transcriptional promoters were used for the expression of BDNF and LuxR, respectively. The final solution conditions exploited substantially less of each molecular component. For example, 66% less amino acid and 33% less of the energy regeneration solutions were used in comparison to commonly used conditions (table S1) (*20*). Other optima within chemical space are likely, as revealed recently by machine learning and high-throughput screening (*21*).

### Artificial cells guide the differentiation of neural stem cells

**Fig. 2:**
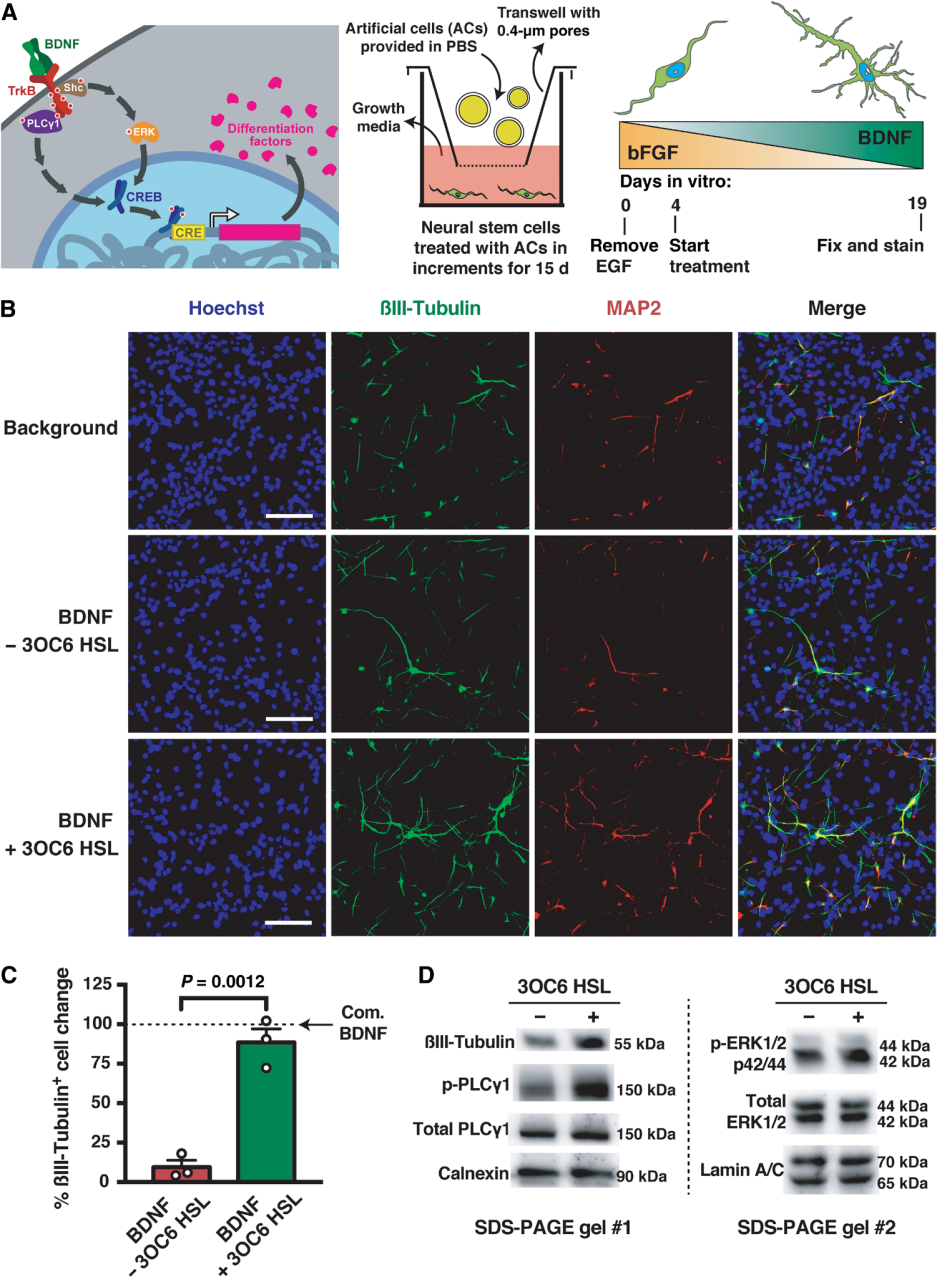
Artificial cells drive neuronal differentiation. (**A**) Overview of artificial cell treatment and mNS cell differentiation strategy. Left: Overview of signaling between artificial cells and mNS cells. Middle: Artificial cells were incubated with mNS cells for 15 days. Artificial cells were washed away, and fresh artificial cells in fresh medium were added every 24 hours. The cartoon represents a cross-section of the well. Right: Over the course of artificial cell treatment (days 4 to 19), BDNF-secreting artificial cells were gradually increased. (**B**) Representative immunostaining microscopy of the differentiation of mNS cells into mature neurons at 19 days. Biologically inactive artificial cells (sfGFP secreting, “Background”) were used for normalization. (**C**) Statistical analysis of βIII-tubulin–overexpressing mNS cells. Cultures treated with commercial BDNF (Com. BDNF) were taken as a reference (100% active, dashed line). The data from (B) were used to generate the plot. Raw data are in fig. S4 (F and G). (**D**) Western blot of predifferentiated mNS cells in response to treatment with artificial cells at 19 days. Scale bars, 50 μm. Data show mean ± SEM for *n* = 3 biological replicates, independent experiments. Statistical test was Student’s *t* test (unpaired, two-tailed). See the Supplementary Materials for detailed figure legend.

Artificial cells built as in Fig. 1 were capable of influencing the differentiation and maturation of mouse embryonic stem cell–derived neural stem (mNS) cells. Over 19 days, the artificial cells were exchanged every 24 hours so as to meet the increasing demand of BDNF during the differentiation of mNS cells. Therefore, old artificial cells were continuously removed, and fresh artificial cells were continuously provided. At the end of this 19-day in vitro protocol (Fig. 2A), the mNS cells showed clear signs of differentiation into neurons, as revealed by the increase in percentage of βIII-tubulin–overexpressing neurons (Fig. 2, B and C). The efficiency of neural differentiation was determined by neuronal cell counting by immunostaining for the pan-neuronal and mature neuronal markers βIII-tubulin and microtubule-associated protein 2 (MAP2), respectively. The overlapping MAP2 and βIII-tubulin signals confirmed the specificity of βIII-tubulin immunostaining and tagging of mature neurons (Fig. 2B and fig. S4A). Conversely, when the artificial cells were not induced to form pores with 3OC6 HSL, the background differentiation levels stayed the same as when incubated with artificial cells that did not synthesize BDNF (±1% difference). Similar background levels of differentiation were observed when only PBS was added to the differentiation medium (21 ± 3% of the overall population). Furthermore, the BDNF released by the artificial cells inhibited apoptosis (fig. S4, B and C), consistent with the biological activity of the synthesized signaling molecule (*22*). If exposed to toxic conditions during neuronal differentiation, mNS cells could give rise to non-neuronal cells, i.e., astrocytes (*23*). However, no such effect was observed (fig. S4, D and E).

If the functionality of the artificial cells was due to the synthesis and release of BDNF, then it should be possible to detect the activation of BDNF-responsive signaling pathways in neural stem cells. To this end, cultures of mNS cells were differentiated for 18 days in the presence of artificial cells and analyzed for activation of tropomyosin receptor kinase B (TrkB)–BDNF signaling. Differentiation into neurons and the phosphorylation of signaling pathway proteins were evaluated by immunoblotting for βIII-tubulin, phospho–phospholipase Cγ1 (PLCγ1), and phospho-ERK1/2^MAPK^ on the 19th day (Fig. 2, A and D). The release of BDNF from the artificial cells induced an increase in phosphorylated PLCγ1 and ERK1/2^MAPK^ (normalized to total PLCγ1 and total ERK1/2^MAPK^) (Fig. 2D). βIII-Tubulin, phospho-PLCγ1, and phospho-ERK1/2^MAPK^ were found to increase by 1.8-, 2-, and 1.5-fold, respectively. The data were consistent with the differentiation of mNS cells resulting from the stimulation of TrkB and the activation of downstream pathways. Together, artificial cells guided the differentiation of neural stem cells into mature neurons in response to an environmental signal.

### Artificial cells communicate with engineered HEK293T cells

**Fig. 3:**
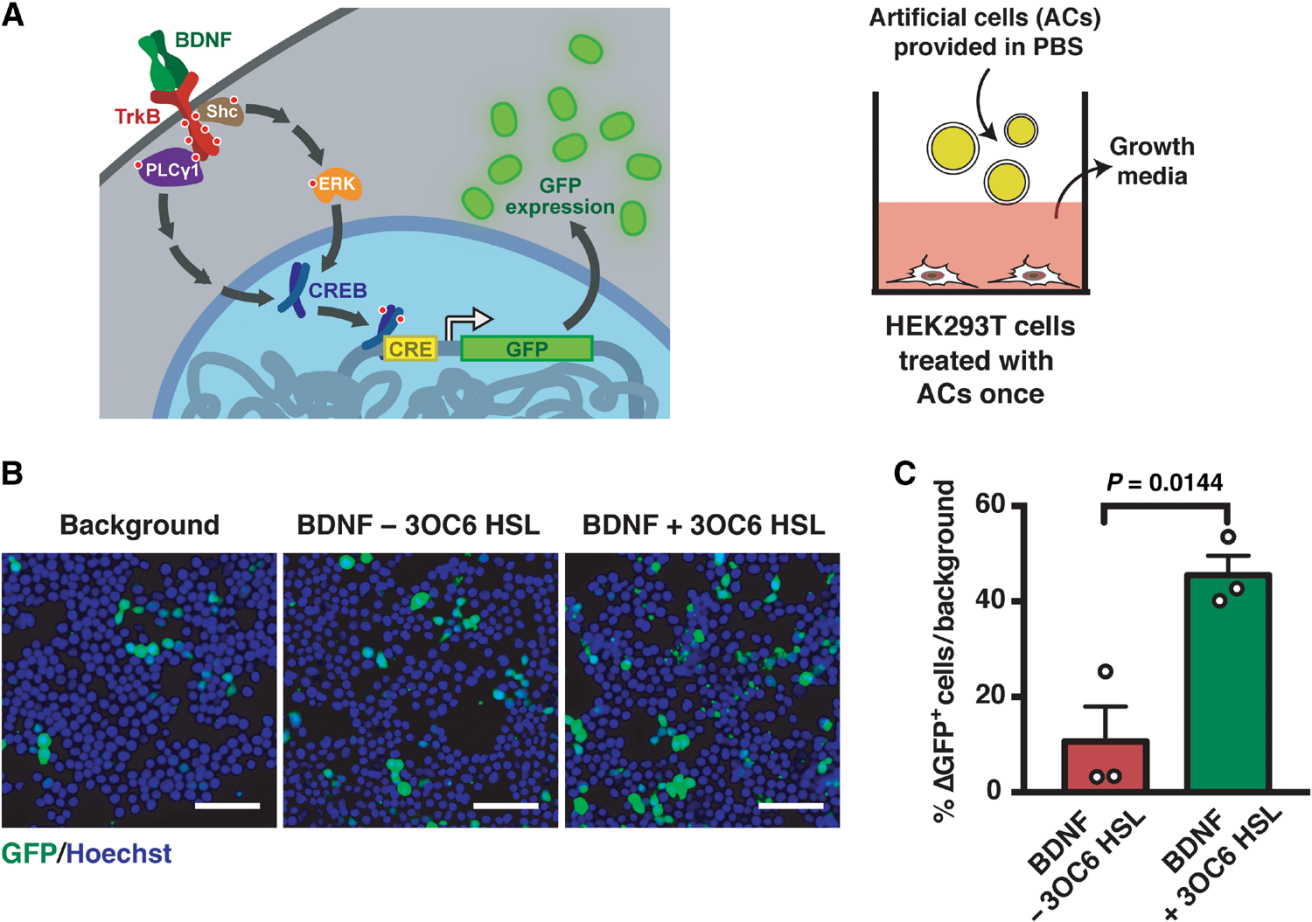
Artificial cells influence the behavior of HEK293T cells. (**A**) Overview of artificial cell treatment of HEK293T cells. Left: Overview of signaling between artificial cells and neural stem cells. Artificial cells activate the expression of genes behind a CRE-regulated promoter, which was GFP. Right: Cartoon representation of the treatment and a cross-section of a cell culture well. (**B**) Representative microscopy images of GFP expression in genetically engineered HEK293T cells. As in Fig. 2, biologically inactive artificial cells were considered as a mock treatment and used for normalization to assess the signal change (indicated as “Background”). Scale bars, 50 μm. (**C**) Statistical analysis of GFP-expressing HEK293T cells treated with artificial cells. Change in number of GFP-expressing cells was calculated using background levels as 0%. The percent difference was calculated as a per capita change in GFP^+^ cells over the entire population with respect to the background. Data show mean ± SEM for *n* = 3 biological replicates, independent experiments. Statistical test was Student’s *t* test (unpaired, two-tailed).

The functionality of the artificial cells was further confirmed with a HEK293T cell line that was engineered to express GFP in response to BDNF (Fig. 3A). The cell line overexpressed the BDNF receptor TrkB and was designed to respond to increased levels of phosphorylated CREB [cyclic adenosine monophosphate (cAMP) response element–binding protein] (fig. S5). The activation of CREB by phosphorylation was expected through TrkB-BDNF signaling, leading to the transcriptional activation of genes under the control of a CRE promoter, in this case GFP (*24*). In other words, GFP expression would only be detected if the artificial cells synthesized and released BDNF in response to 3OC6 HSL. Because of the polyclonal nature of the final cell line, varying levels of GFP expression were expected upon induction (*25*). To better assess the changes in expression of GFP, HEK293T cells were analyzed at a population level. Compared to the negative control, where artificial cells produced and secreted an inert protein, a 45 ± 4% increase in the number of HEK293T cells that expressed GFP was detected when the chemical trigger 3OC6 HSL was added (Fig. 3, B and C). Conversely, incubation with artificial cells that did not express protein pores resulted in only a 10 ± 7% increase of the fluorescent cell population (Fig. 3C). Together, artificial cells were capable of eliciting the desired phenotypic changes in eukaryotic cells through the controlled release of BDNF.

### The component parts of the artificial cells are functional

**Fig. 4:**
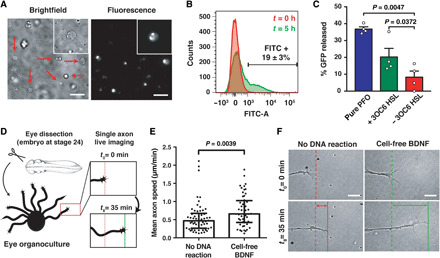
Characterization of artificial cells and cell-free synthesized BDNF under physiological conditions. (**A**) Representative microscopy images of artificial cells that synthesize a BDNF-sfGFP fusion protein at *t* = 5 hours. Arrows indicate artificial cells. Scale bars, 10 μm. (**B**) Representative flow cytometry of BDNF-sfGFP–producing artificial cells. (**C**) GFP release from artificial cells. Recombinant PFO, denoted as “pure PFO,” was provided at a final concentration of 4 μM (0.2 μg/μl). Data show mean ± SEM for independent experiments, *n* = 4 biological replicates. Statistical test was Student’s *t* test (paired, two-tailed). (**D**) Experimental overview of the single axon live-imaging strategy of *Xenopus laevis* ex vivo eye organocultures. (**E**) An increase of axon speed was observed when cell-free expressed BDNF was supplied. (**F**) Representative images of RGC axons from real-time imaging. Scale bars, 10 μm. Data show median with interquartile for *n* = 4 independent biological replicates. Statistical test was two-tailed Mann-Whitney test, where the data were not normally distributed (Shapiro-Wilk test). At least 55 growth cones were counted for each group.

To ensure that the component parts of the artificial cells functioned under physiological conditions as intended, we sought to confirm protein expression within the vesicles. The intravesicular production of genetically encoded superfolder GFP (sfGFP; fig. S6, A to C) and a BDNF-sfGFP chimera (Fig. 4, A and B) was assessed by fluorescence imaging and flow cytometry. After 5 hours, 19 ± 3% of the artificial cells produced detectable levels of BDNF-sfGFP (Fig. 4B). Comparison to a standard curve showed robust expression, with an intravesicular concentration of ca. 65 ng/ml (fig. S6, D and E). The fact that roughly one in five artificial cells were active under physiological conditions confirmed the functionality of our system, particularly when considering the complexity of encapsulating the large number of components needed for cell-free translation.

Next, we sought to characterize the formation of functional pores within the artificial cells. First, the cell-free expression of functional PFO pores was confirmed by quantifying the release of fluorophore by spectrofluorometry, flow cytometry, and size exclusion chromatography (figs. S7 and S8). Co-encapsulation of the cell-free expression system with purified GFP led to the regulated release of the fluorescent protein from the artificial cells, confirming that cell-free expressed PFO could release globular proteins (Fig. 4C and fig. S8). After 16 hours in the presence of 3OC6 HSL, a nearly threefold increase in GFP escape from the artificial cells was observed with respect to artificial cells in the absence of 3OC6 HSL (Fig. 4C and fig. S8D). The fraction of artificial cells that was capable of synthesizing protein (Fig. 4B and fig. S6) was consistent with the fraction of artificial cells that released cargo via the activity of expressed PFO (Fig. 4C and fig. S8D). Titrations with recombinantly expressed and purified PFO were consistent with the formation of functional pores with less than 30 nM protein (fig. S8E).

### Artificial cells are stable under physiological conditions

Release of BDNF was due to the activity of PFO and not due to the degradation of the artificial cells. The integrity of the artificial cells was not disrupted after 24 hours at either 30° or 37°C, as judged by the leakage of encapsulated dextran and pyranine (figs. S9 and S10). The artificial cells could withstand culture medium of low osmolality, i.e., L-15 (fig. S9), Dulbecco’s modified Eagle’s medium (DMEM) (figs. S9 and S10), and DMEM supplemented with 10% (v/v) fetal bovine serum (FBS) (fig. S11). Nevertheless, as a precautionary measure, old artificial cells were removed and replaced with fresh artificial cells every 24 hours (Fig. 2A). After 1 week, 85% of the artificial cells survived coincubation with HEK293T cells under serum-deprived conditions (fig. S12). Cell-free translation inside of artificial cells typically ceases after 5 hours (*26*) and likely halted earlier when expressing PFO due to the leakage of the substrates needed for protein synthesis. However, the S12 extract that supported the synthesis of mRNA and protein was durable, retaining 50% activity after 6 hours of dormancy at 37°C (fig. S13). The stability data (figs. S9 to S13) and the data demonstrating that PFO pores were capable of releasing entrapped macromolecules (fig. S8, D and E) were consistent with artificial cells that released BDNF upon induction with 3OC6 HSL and not because of decomposition of the membrane. Furthermore, the artificial cells were not toxic to HEK293T cells. No toxicity was observed with 3-(4,5-dimethylthiazol-2-yl)-2,5-diphenyltetrazolium bromide (MTT) assay after coincubation for 24 hours and 1 week (fig. S14). The toxicity data with HEK293T cells (fig. S14A) and the functionality data with artificial cells (fig. S8E) together indicated that the artificial cells produced and/or leaked PFO in the nanomolar range. While more work would be needed to convert such artificial cells into a technology that could be used in clinical trials, the current formulation is robust and functional under physiological conditions. We further note that the liposomal delivery of drug molecules currently exists (*27*), and the incorporation of PEGylated lipids increases circulation time (*28*). The artificial cells described here contained 1 mol % PEGylated lipid.

### Cell-free expressed BDNF is active

Activity was due to the synthesis and release of BDNF and not due to other components of the artificial cell. Cell-free expressed BDNF elicited similar phenotypic changes as observed with BDNF-secreting artificial cells and additionally showed activity ex vivo with embryo-derived organocultures (Fig. 4, D to F; fig. S15; and movies S1 and S2). For example, both commercial and cell-free expressed BDNF triggered the TrkB signaling pathway and induced the expression of GFP in engineered HEK293T cells (figs. S5E and S15, A to C). In parallel to the increase in GFP expression, elevated levels of the signaling protein ERK1/2^MAPK^ were detected upon induction with BDNF (figs. S5C and S15C). Cell-free expressed BDNF also promoted the differentiation and survival of neurons in vitro with mNS cells. After 13 days in vitro, the cell-free BDNF-treated mNS cells more extensively differentiated into neurons in comparison to the negative control, which consisted of the cell-free reaction mixture without the DNA template encoding BDNF (fig. S15, E and F). Furthermore, cell-free expressed BDNF protected against apoptosis, as assessed by cell counting of immunostained cells for the cleaved form of caspase-3 (fig. S15, E and G). Last, the ability of cell-free expressed BDNF to promote neuron differentiation ex vivo was gauged by evaluating the acute activity of BDNF on axon elongation. Using a single axon live-imaging strategy (Fig. 4D), the bath application of cell-free expressed BDNF elicited a 40% increase in outgrowth velocity within a 35-min exposure (Fig. 4, E and F). Because of the high sensitivity of ex vivo axons to the concentration of BDNF, it was not possible with the current formulation of artificial cells to set up a meaningful analogous experiment. Nevertheless, for all three model systems, the data collected with cell-free expressed BDNF were comparable to those of commercial BDNF (Fig. 4E and fig. S15).

The optimized *E. coli* S12 reaction was capable of producing biologically relevant quantities of BDNF. In vitro concentrations of cell-free expressed BDNF were determined by immunoblot quantification. With total cell-free expressed BDNF concentrations being 100 ± 20 μg/ml, at least 5 to 10% of the total soluble protein (10 ± 2 μg/ml) was found to be in the active, dimeric state (fig. S16F). Encapsulation led to a 3.5-fold reduction in the amount of synthesized protein in comparison to batch reactions (fig. S13C). Following the assembly of the artificial cells, only 10% of the initially encapsulated cell-free reaction was estimated to have been retained, which corresponded to a volume of 1 μl. The internal volume of all of the artificial cells added together was approximately ^1^/_500_th of the volume of the growth medium, taking into account losses due to handling. Because 20% of the synthesized protein was released from the artificial cells (Fig. 4C), the final concentration of BDNF was 2500-fold lower. However, the exploited differentiation protocol increased the amount of fresh artificial cells that were added every 3 days by 50%. Therefore, the concentration of released BDNF was conservatively estimated to be 50 ± 10 ng/ml over the course of the entire mNS cell differentiation protocol. This value was in agreement with the sfGFP produced inside of the vesicles (ca. 65 ng/ml; fig. S6), earlier reports by others on protein synthesis in vesicles (*29*), and previously optimized differentiation protocols (*22*). Therefore, physiologically relevant concentrations of BDNF could be produced and released from the artificial cells to elicit a response from eukaryotic cells.

## DISCUSSION

The ability of artificial cells to synthesize and release molecules on demand will likely provide for numerous possibilities to influence biological systems in a safe and effective way (*26*, *30*). A genetically programmable system that is similar to what was described here could be envisaged to integrate within the gut-brain axis by exploiting the naturally occurring quorum signaling pathways between bacteria (*31*, *32*). Further advances could come from improvements to the sensing capabilities of the engineered system so that artificial cells could respond to the physiological changes of the host, e.g., changes to the concentration of cations (*33*–*35*), neurotransmitters (*36**)*, or other molecules and factors present in the extracellular matrix (*37*, *38*). To do so, it will be important to devise systems that can carry out translation for greater than 5 hours, perhaps by harnessing the nutrients of the host. Tissue-like materials (*39*) composed of three-dimensional printed artificial cells (*40*) could be engineered to aid in the healing of damaged tissue through stem cell differentiation and axon regeneration, particularly for spinal cord injury (*41*). Tissue-targeted delivery could be achieved by displaying surface receptors (*27*) so that artificial cells could be engineered to produce and release eukaryotic signaling peptides (*28*, *42*, *43*). Once designed and decorated, such artificial cells could integrate within the host to controllably release extracellular factors and small molecules (*26*) or to deliver gene editing machinery (*44*, *45*).

This work brings the field closer to the construction of artificial cells that go beyond what is typically envisioned for smart drug delivery systems (*46*) and opens up new possibilities in theranostics and therapeutics (*26*). Artificial cells are capable of more than functioning as simple drug delivery vehicles. With the technologies described here as a platform, it should be possible to develop artificial cells that regularly monitor physiological conditions and, in response, synthesize and release different drug molecules. In this way, the changing needs of the host would be rapidly met in a manner that does not flood the entire organism with drug molecules.

## MATERIALS AND METHODS

### Materials and reagents

All chemicals were purchased from Sigma-Aldrich (Merck) with highest possible purity, unless otherwise noted. Commercial BDNF was recombinantly expressed in *E. coli* and purchased from PeproTech. Lipids were from Avanti Polar Lipids. *E. coli* Rosetta2 (DE3) was used for the preparation of the S12 extract. *E. coli* NEB5α (New England Biolabs) was used for general molecular cloning purposes. *E. coli* BL21(DE3) (New England Biolabs) was used for recombinant protein expression.

### Genetic constructs and recombinant proteins

All cloning was by Gibson Assembly (*47*), unless otherwise noted. For all constructs used with artificial cells, pSB1A3 from the iGEM registry (parts.igem.org) was the vector backbone. Promoter, terminator, and short tag (<50 base pairs) sequences were taken from the iGEM registry (BBa_R0040, BBa_J23101, BBa_J23106, BBa_J23117, BBa_R0062, and BBa_B0010). An *E. coli* codon-optimized sequence of mature murine BDNF was synthesized by GenScript. Human TrkB complementary DNA (cDNA) was synthesized by GenScript in two parts, joined in-house by Gibson Assembly, and cloned into a pLenti vector with standard molecular cloning techniques. The LuxR gene was taken from the registry plasmid BBa_T9002. The gene for PFO was from a previously published construct (*48*). CRE-regulated gene expression plasmid was taken from a previous report (*25*). The exploited genetic constructs and plasmids used in this study are reported in table S2. Recombinant proteins were expressed and purified as previously described (*49*). An N-terminal 6xHis-tag was used with the flexible linker sequence (GGGS)_1–2_.

### Preparation of *E. coli* S12 extract

Homemade *E. coli* S12 extract was prepared using published protocols as a guide (*20*). Rosetta2 (DE3) was streaked on 2xYT+P agar plates containing chloramphenicol (34 μg/ml) and incubated overnight at 37°C. A single large colony was picked for an overnight (precisely 15 hours) culture to prepare starter freezer stocks (10× concentrated) in 2xYT+P, containing 12.5% glycerol, 100 mM MgSO_4_, and 25 mM tris-Cl (pH 8.0). To prepare the *E. coli* S12 extract, a new overnight (15 hours) culture was prepared from the freezer stock in 2xYT+P with chloramphenicol (34 μg/ml). The next day, the culture transferred to 1-liter prewarmed 2xYT+P in 5-liter flasks with 1:100 dilution and incubated with shaking (220 rpm) at 37°C without disturbing for 3 to 3.5 hours. The cells were then immediately harvested and centrifuged for 10 min at 6000*g*, 4°C. From this point on, the cells were constantly kept at 4°C and all centrifugations were performed under identical conditions. The bacterial pellets were briefly washed, resuspended in prechilled S12A buffer [14 mM Mg^2+^ glutamate, 60 mM K^+^ glutamate, 2 mM dithiothreitol (DTT), 50 mM tris-Cl (pH 7.7) adjusted with concentrated acetic acid], centrifuged again under the same conditions, and resuspended in ^1^/_10_th of the original S12A buffer volume to transfer the cells to prechilled 50-ml Falcon tubes. Following a second round of washing, the residual buffer was removed, and the wet pellet weight was determined. Autoclaved glass beads (5 times of total weight, 100 μm diameter) and S12A buffer (0.9 times of total weight) were then added to the cell pellet in three rounds with vortexing after each addition. Falcon tubes were then cut in half, and the slurry-bead mixture was carefully transferred to bead beating tubes using 1-ml syringes without a needle. Care was used to avoid visible bubbles inside the tubes, and the resulting mixture was stored overnight at −80°C. After thawing the slurry-bead mixture on ice for <2 hours, bead beating was performed at a beat rate of 6.5 m/s for 30 s (×2) (FastPrep-24, MP Biomedicals). Then, the crude cell extract was separated from the glass beads by centrifugation with Bio-Rad Bio-Spin columns. The cell extract was transferred to new tubes to remove the cellular debris, requiring at least two centrifugations. The crude cell extract was incubated at 37°C with shaking (220 rpm) for 80–90 min in open-capped tubes and subsequently clarified at least twice by centrifugation. Last, the extract was dialyzed against freshly prepared S12B buffer (14 mM Mg^2+^ glutamate, 60 mM K^+^ glutamate, 1 mM DTT, brought to pH 8.2 with 2 M tris-base). The first round of dialysis was for <3 hours. The second round of dialysis was run overnight (<24 hours) at 4°C. Dialysis exploited a 10-kDa MWCO SnakeSkin dialysis tube (Thermo Fisher Scientific) with gentle stirring. Following dialysis, the extract was aliquoted, stored at −80°C, and used within 1 year from the preparation date.

### In vitro transcription-translation

In vitro and artificial cell transcription-translation reactions were performed at 30°C in a final volume of 10 to 50 μl. In vitro reactions were either in a Rotor-Gene Q qPCR machine (Qiagen) or in a plate reader without shaking (Tecan Infinite M200). Artificial cell reactions were in an incubator. For plate reader experiments, each well in black/clear bottom 96-well plate (Costar 3603) was covered with 50-μl sterile mineral oil to avoid evaporation. For *E. coli* S12 reactions under physiological conditions, the final composition of amino acid solution was 0.5 mM each. The energy solution was 20 mM 3-phosphoglyceric acid, 1 mM adenosine triphosphate (ATP), 1 mM guanosine triphosphate (GTP), 0.6 mM cytidine triphosphate (CTP), 0.6 mM uridine triphosphate (UTP), transfer RNA (tRNA) (0.133 mg/ml), 0.17 mM coenzyme A, 0.22 mM nicotinamide adenine dinucleotide (NAD^+^), 0.5 mM cAMP, 0.045 mM folinic acid, 0.66 mM spermidine, and 33 mM Na^+^ Hepes (pH 8.0). The supplement solution was 2% (w/v) polyethylene glycol 4000 (PEG 4000), 10 mM Mg^2+^ glutamate, 10 mM K^+^ glutamate, 20 mM sucrose, and 6 mM maltose. Table S1 summarizes the final concentrations of the optimized system. For the comparison between batch-mode and water-in-oil emulsion expression, the reactions were either encapsulated inside of the emulsions following incubation in vitro or directly initiated within the emulsion. The mineral oil–dissolved lipids for the water-in-oil emulsions were prepared as described in the next section and contained POPC supplemented with 1% (v/v) sorbitan monooleate (Span 80). Plasmid DNA was obtained using Qiagen Plasmid Midi kits (12145) and resuspended in nuclease-free deionized water. The DNA was quantified with NanoDrop 2000c (Thermo Fisher Scientific), diluted, aliquoted, stored at −20°C at a concentration of 200 nM, and used at a final concentration of 10 to 40 nM. Reaction components were assembled in the following order with brief vortexing after each addition and without centrifugation: *E. coli* S12 extract, amino acid solution, energy solution, PEG, supplement solution, water, and plasmid DNA.

### Generation of artificial cells

Vesicles and artificial cells were generated using the previously published conversion of water-in-oil emulsion to vesicle methodology with slight modifications (fig. S3) (*50*). Generally, solutions of equal osmolality for the outer [either alanine or Dulbecco’s PBS (DPBS)] and inner (*E. coli* S12 reaction or sucrose) vesicle solutions were used. Osmolality was determined by freezing-point depression of 25-μl samples with an osmometer from Löser. Vesicles were made with POPC and cholesterol (Sigma-Aldrich) at a total final concentration of 380 μM, either with or without 1 mol % 1,2-distearoyl-*sn*-glycero-3-phosphoethanolamine-*N*-[biotinyl(polyethylene glycol)-2000] (ammonium salt) [DSPE-PEG(2000) Biotin, Avanti Polar Lipids]. Vesicles were prepared in sterilized glass vials using sterile cell culture–grade mineral oil (BioXtra 5310, Sigma-Aldrich) and protected from light either by wrapping in aluminum foil or by using amber vials. Briefly, chloroform lipid stocks were pipetted into glass vials and carefully dried under constant flow of N_2_. The resulting dry lipid film was put under vacuum for >4 hours to remove residual solvent. The dried lipid was dissolved in mineral oil, vortexed, and briefly incubated at 80°C in an oven multiple times until the undissolved material was no longer visible. The dissolved lipids were sonicated in a water bath (50° to 60°C) for 30 to 60 min, depending on the vial size and glass thickness. The lipids were then stored in the dark and used within 1 week after preparation. The resulting lipid solution was carefully overlaid onto an outer solution in an Eppendorf tube. The mixture was left undisturbed for 30 to 60 min to allow phase separation and the formation of a monolayer (fig. S3). Then, 10 μl of freshly assembled *E. coli* S12 reaction was pipetted into 500 μl of mineral oil–lipid solution in a separate tube to generate a water-in-oil emulsion by agitation by rapidly drawing the tube over a rack. The emulsion was left to equilibrate at room temperature for ca. 1 min and overlaid onto the interface, followed by 2 to 3 min of incubation. The artificial cells were formed and pelleted by centrifugation at 5000*g* for 5 min. The upper mineral oil layer was removed by vacuum until ca. 50 μl of aqueous solution remained. The artificial cell pellet was washed at least two times (1 ml each) with either alanine or DPBS without Mg^2+^ and Ca^2+^ (for cell culture experiments) and with centrifuging at 5000*g* for 1 min. Following this workup, each 1.5-ml sample of artificial cells contained at most 0.5 to 1 μl of encapsulated cell-free reaction.

### Calcein release assay and PFO pore functionality

The preparation of vesicles followed previously published protocols (*51*, *52*). The non-S12 reaction–encapsulating (“empty”) vesicles with different cholesterol content were prepared by thin lipid film hydration with Milli-Q water to 100 μl. The total lipid concentration was 23 mM (POPC + cholesterol). The hydrated lipid mixture was subjected to five freeze/thaw cycles. The empty vesicles were homogenized with a handheld homogenizer (IKA T10 Basic) and extruded to 100 nm with a mini extruder from Avanti Polar Lipids. The vesicles were then lyophilized overnight with Rotavap R-210 (Büchi) or CentriVap DNA concentrator (Labconco) and stored at −20°C. The lyophilized stocks were used within 1 year of preparation. To test for the efficiency of pore formation by PFO, the vesicles were rehydrated with 80 mM calcein and purified from unencapsulated fluorophore by size exclusion chromatography with sepharose 4b resin. Ten microliters of the in vitro transcription-translation reaction was assembled as described above (see the “In vitro transcription-translation” section) with freshly purified calcein-containing vesicles with at least ^1^/_10_th of final reaction volume (1 μl). The reaction was incubated at 30°C, and fluorescence (λ_ex_ = 485 nm; λ_em_ = 515 nm) was monitored over time with a RotorGene Q instrument. For the tests of activity of PFO expressed within artificial cells, a plasmid DNA encoding LuxR-PFO (DT034A) was encapsulated at a concentration of 20 nM. Expression of PFO was induced after 30 to 60 min of incubation at 30°C with 10 μM 3OC6 HSL from a 1 mM stock in 10% (v/v) dimethyl sulfoxide (DMSO). The reaction was stopped 16 hours after induction. The artificial cells were then treated and analyzed as described in the next section.

### Flow cytometry

Artificial cells were diluted in either 300 mM alanine or PBS before analysis. Flow cytometry was with a FACSCanto A (BD Biosciences) with a flow rate setting of “low” and voltage values of 350 V (for SSC-W), 400 V [SSC-A, FSC-A, and FITC (fluorescein isothiocyanate) for Alexa Fluor 488–dextran and pyranine (8-hydroxypyrene-1,3,6-trisulfonic acid)], or 700 V (FITC for GFP and sfGFP). For each run, at least 10,000 gated events or 50,000 total events were recorded with a gating strategy that took into account only fractions that contained giant vesicles (fig. S3D) (*53*, *54*). The flow cytometer was calibrated with 1- and 10-μm particles for voltage optimization and particle size determination. Single and double events were discriminated by SSC-A versus SSC-W plots, and only single events were taken into account for statistical analyses (fig. S8B). For the experimental gating strategy, the “No DNA” and/or “No dextran/GFP” samples were used as negative controls to determine the FITC-positive events to create a new gate. Both the loss of FITC intensity (due to GFP escape through PFO pores) and percent changes in events of specific gates were taken into account for statistical analysis. The data were analyzed with either FACSDiva software (BD Biosciences) or FlowJo v10 (FlowJo LLC). All events were plotted in pseudocolor.

### Microscopy

Artificial cells were imaged with an Axio Zeiss Observer Z1 microscope equipped with either a 100× (Plan-Apochromat 100x/1.4 oil DIC) or 40× (EC Plan Neofluar 40×/0.75) objective. Water-in-oil emulsions were imaged with 20X (LD Plan Neofluar 20×/0.4 Korr Ph2 M27) objective. For both, the excitation and emission values were λ_ex_ = 484 ± 25 nm and λ_em_ = 525 ± 50 nm. Artificial cell images were acquired with a physical spacer between the coverslip and glass slides that were treated with Repel Silane (GE Healthcare). For HEK293T and/or stem cells, the imaging was performed with either an Axio Zeiss Observer Z1 (objectives: 10× EC, enhanced contrast; 20× LD, long distance) or a Nikon Eclipse Ti2 spinning disc confocal microscope. HEK293T cells were cultured with 15-mm glass coverslips pretreated with poly-l-lysine (100 μg/ml) (Sigma-Aldrich) and imaged with glass slides prepared with homemade mounting medium with the following recipe: 4 mM Mowiol (molecular weight: 31,000; Sigma-Aldrich), 25% glycerol (v/v), 0.3% (w/v) sodium azide, and 50 mM tris-Cl (pH 8.0). For nonbiased data collection, all images were taken with either an automated stage (Nikon Eclipse Ti2) or a random field of the culture, only focused/determined by nuclei (Observer Z1). For the *Xenopus* collapse assays, images of growing axons were taken in brightfield with an Axio Zeiss Observer microscope equipped with a 40× objective. For the collapse assay, the coverslips were mounted with ImmunoHistoMount (Sigma-Aldrich) on a glass slide and observed with a Leica DMi8 microscope equipped with an HCX PL Fluotar L 40×/0.6 Leica DMi8 objective.

### HEK293T cell culture

Wild-type HEK293T cells were obtained from the American Type Culture Collection (ATCC) cultured in high-glucose (4.5 g/liter) complete DMEM (Corning) with 10% (v/v) FBS (Gibco), penicillin/streptomycin (100 μg/ml) (Corning), and 2 mM l-glutamine (Corning) at 37°C with 5% CO_2_. The production of lentivirus was performed using previously established protocols (*55*). Briefly, the viruses were produced in wild-type HEK293T with a second-generation lentiviral system and transfected with pLenti–cytomegalovirus (CMV)–TrkB and auxiliary viral packaging plasmids using Lipofectamine 2000 (Thermo Fisher Scientific) according to the manufacturer’s instructions. After transfection, the medium was changed following overnight incubation at 37°C. Subsequently, 24, 48, and 72 hours later, the media were pooled and concentrated using 50% (w/v) PEG 4000. For the generation of a CMV-TrkB stable cell line, wild-type HEK293T cells were seeded at a cell density of 2 × 10^6^ in 10-cm dishes and transduced at 70% confluency. On day 3, the cells were challenged with puromycin (1 μg/ml) and maintained in culture until clear single colonies were observed (fig. S5A). Single colonies were isolated, picked, and transferred to new six-well plates to propagate, while selection was kept until the expression levels were verified by immunofluorescence against total TrkB protein (fig. S5B). For the CMV-TrkB-CRE-GFP cell line, cells were transfected with plasmid DNA containing SV40 T-antigen using polyethylenimine (PEI; 1 mg/ml, Sigma-Aldrich) with a plasmid backbone linearized with ScaI. On day 2, cells were challenged with zeocin (400 μg/ml) (Thermo Fisher Scientific) and maintained in culture until clear single colonies appeared (fig. S5D). The resulting stable polyclonal cell line was used in all subsequent experiments, unless otherwise noted. Because of the polyclonal nature of the final cell line, varying levels of GFP expression upon induction were expected, as reported previously (*25*). Cells were confirmed as mycoplasma negative.

### Functionality of cell-free expressed BDNF with HEK293T

For Western blotting, HEK293T-CMV-TrkB cells were seeded in 12-well plates at a density of 2 × 10^5^ per well and cultured at 37°C until reaching 70% confluency. On the day of treatment, the cells were washed once with warm DPBS without Mg^2+^ and Ca^2+^ (Gibco) and then serum-starved for 4 hours with complete DMEM without serum. Cell-free BDNF was first diluted 1:3 with DPBS without Mg^2+^ and Ca^2+^ and then diluted further to 1:1000. The cells were incubated for 2 to 30 min at 37°C; lysed with buffer containing 50 mM Hepes (pH 8.0), 2% (v/v) SDS, and 50 mM DTT; and placed on ice or stored at −20°C until use for immunoblotting. For fluorescence imaging, the day before treatment, HEK293T-CMV-TrkB-CRE-GFP cells were seeded into poly-l-lysine–treated (37°C for 1 hour with [final] = 0.1 mg/ml) 24-well plates with a density of 2 × 10^5^ per well. On the day of the treatment, the cells were washed once with complete DMEM without FBS (1 ml) and incubated at 37°C for ca. 7 hours until the treatment (500-μl final volume). The cell-free synthesis of BDNF was as described in the “In vitro transcription-translation” section. After 7 to 12 hours of cell-free expression at 30°C, the BDNF-containing solution was diluted 1000- or 500-fold (from 4-fold predilution) with PBS, before addition to the HEK293T cells. Subsequently, the cells were handled as described in the “Immunofluorescence” section (without blocking or the addition of antibody).

### Communication between artificial cells and HEK293T cells

Eukaryotic cells were prepared as described in the previous section for fluorescence imaging. The only exception is that the cells were seeded on poly-l-lysine–treated 24-well plates (0.1 mg/ml, Sigma-Aldrich). Artificial cells of the same experimental group were prepared in four identical 1.5-ml tubes, concentrated into single vials, and incubated at 30°C for 5 hours and added on top of the eukaryotic cells (without Transwell). The artificial cell and eukaryotic cell samples were coincubated at 37°C for an additional 16 hours. Subsequently, the HEK293T cells were washed twice with 500 μl of PBS to remove the artificial cells. From this point on, the eukaryotic cells were handled as described in the “Immunofluorescence” (without blocking or the addition of antibody) and “Microscopy” sections.

### Neural stem cell culture

Mouse neural stem cells (mNS) were derived from murine embryonic stem cells as previously described (*22*). mNS cells were routinely passaged every 3 to 4 days at a ratio of 1:3 to 1:5 in mNS self-renewal medium composed of Euromed-N (Euroclone), supplemented with N2 supplement [1% (v/v), Thermo Fisher Scientific], EGF (epidermal growth factor) and bFGF (basic fibroblast growth factor) (both 20 ng/ml, PeproTech), and GlutaMAX (2 mM, Thermo Fisher Scientific). For passaging, mNS cells were incubated with StemPro Accutase (Thermo Fisher Scientific) and centrifuged at 260*g* for 3 min. The pellet was resuspended in fresh medium and plated onto culture-treated plastic vessels.

For neuronal differentiation, mNS cells were exposed to conditions described in the literature (*23*). Briefly, 1 × 10^5^ cells/cm^2^ were seeded onto culture-treated plastic vessels in D1 medium composed of Euromed-N (Euroclone) medium supplemented with N2 supplement [1% (v/v), Thermo Fisher Scientific], B27 supplement [0.5% (v/v) Thermo Fisher Scientific], bFGF (10 ng/ml, PeproTech), and GlutaMAX (2 mM, Thermo Fisher Scientific) and cultured for 72 hours (0 to 3 days in vitro). No BDNF was added during this period. Cells were then gently detached as above and reseeded on laminin-coated (3 μg/ml, Thermo Fisher Scientific) plastic 24-well plates at a density of 3.5 × 10^4^ cells/cm^2^ in D2 medium [1:3 mix of DMEM/F-12 and Neurobasal medium, 0.5% (v/v) N2, 1% (v/v) B27, bFGF (10 ng/ml)] for 96 hours (4 to 6 days in vitro). The day after reseeding (on day 4), either commercial BDNF, artificial cells, or cell-free reaction treatments were started. For final neuronal maturation (7 to 16 days in vitro), the medium was switched to D3 medium [1:3 mix of DMEM/F-12 and Neurobasal medium, 0.5% (v/v) N2, 1% (v/v) B27, and bFGF (6.7 ng/ml)] and renewed every 3 days along with the treatment. Cells were confirmed as mycoplasma negative.

### Functionality of cell-free BDNF with neural stem cells

mNS cells were differentiated as described in the previous section with the following modifications. From the fourth day in vitro, until the end of the differentiation procedure, the cell-free BDNF synthesis reaction was performed as described in the previous section for 7 to 12 hours at 30°C. The cell-free expressed BDNF was provided at increasing concentrations every 72 hours with the following dilutions: 1:1000 (4 to 6 days in vitro), 1:750 (7 to 9 days in vitro), and 1:500 (10 to 19 days in vitro). Commercial BDNF was provided at final concentrations of 20, 30, and 40 ng/ml.

### Communication between artificial cells with neural stem cells

mNS cells were differentiated as in the “Neural stem cell culture” section until the treatment day. Starting from the first day of treatment with artificial cells, 24-well Transwell plates (Costar 3413, 6.5-mm insert, 0.4-μm polycarbonate membrane) with permeable supports were used to avoid the influences of direct contact between the artificial cells and the stem cells. Growth medium was added to the lower chamber (500 μl), and artificial cells (100 μl) were added to the upper chamber. BDNF-producing artificial cells contained DNA encoding BDNF (DT033A) at 20 nM and another plasmid that encoded LuxR and PFO (DT034A) at 10 nM. Every 72 hours, the total amount of artificial cells was increased 1.5-fold in concentration. In all cases, 10 μM 3OC6 HSL was used to induce the expression of PFO. The addition of 3OC6 HSL from the stock solution never gave a final culture concentration of DMSO above 0.1% (v/v). Every 24 hours, 350 μl of medium was replaced with fresh medium and Transwell chambers were thoroughly washed with PBS before supplying fresh artificial cells. From this point on, the cells were handled as described in the “Immunostaining” and “Microscopy” sections. Neural differentiation was stopped at 19 days following observation by microscopy that showed that satisfactory levels of neurons in the cultures were achieved in the reference positive control cultures (i.e., cultures maintained under standard differentiation conditions with commercial BDNF).

### Maintenance of *Xenopus laevis* embryos

*Xenopus laevis* embryos were obtained by in vitro fertilization, raised in 0.1× MMR (Marc’s modified Ringer’s solution) (pH 7.5) at 14° to 22°C, and staged according to previous literature (*56*). All experiments with animals were approved by the Italian Ministero della Salute with the authorization n° 546/2017-PR according to art.31 of D.lgs. 26/2014.

### Analysis of axon elongation

For *Xenopus* retinal explant cultures, glass coverslips (Bellco) or glass-bottom dishes (MatTek) were coated with poly-l-lysine (Sigma-Aldrich, 10 μg/ml diluted in water) and with laminin (Sigma-Aldrich, 10 μg/ml) diluted in L-15 medium (Gibco). Whole eyes from anesthetized embryos were microdissected and cultured at 20°C in 60% L-15 + penicillin-streptomycin-fungizone [1% antibiotic-antimycotic (Thermo Fisher Scientific)] medium. The day before treatment, cell-free BDNF synthesis reactions were performed as described in the “In vitro transcription-translation” section at 30°C for 7 to 12 hours. The cell-free expressed BDNF and negative control reactions (the same cell-free reaction but lacking the DNA encoding BDNF) were provided at a dilution of 1:200. Commercial BDNF (PeproTech) was provided at a final concentration of 50 to 100 ng/ml. All conditions were tested for outgrowth within the same experiment and with identical culture conditions. The imaging and analysis were performed as stated in the “Microscopy” section. A z-stack of 10 to 12 planes of 0.7 μm was acquired every 5 min for 35 min, giving rise to 8 time points in total. The average speed of each axon was calculated as the mean of the velocity measured for each time point. Fiji (ImageJ) software was used to analyze images starting from the raw .czi files. The best plane of focus for each time point was selected. The distance covered by the axon from one time point to the next was traced using the Plugin Tracking-ManualTracking of ImageJ.

### Collapse assay

Retinal ganglion cell (RGC) axon collapse assays were performed as reported in the literature (*57*, *58*). An aliquot (1 to 12.5 μl) of the reactions was mixed with 1× PBS to a final volume of 100 μl and applied for 10 min to RGC axons cultured in 400-μl medium with coverslips as described in the previous section. Before imaging, explants were fixed with 2% (v/v) formaldehyde (Thermo Fisher Scientific) containing 7.5% (w/v) sucrose (prepared in 1× PBS) for 30 min and subsequently washed three times with 1× PBS. Axons were imaged as described in the “Microscopy” section, and collapsed growth cones were counted blind to the observer. Only single axons growing individually from the eye explant were taken into account and analyzed to avoid confounding biological influences because cell-to-cell interactions may provide trophic support for axons. The data were expressed as the percentage of collapsed growth cones to the total number of single growth cones counted. Growth cones were considered as collapsed when the growth cones had no lamellipodium and less than or equal to two filopodia (each less than or equal to 2 μm) (*59*).

### Stability and toxicity of artificial cells

For the analysis of toxicity and stability, the artificial cells were prepared as described in the “Generation of artificial cells” section. An aliquot of artificial cells was either purified on a sepharose 4b column as previously described (*60*) or analyzed by flow cytometry at 1:50 dilution. The same gating strategy was used as described in the “Flow cytometry” section. For the analysis of toxicity, TrkB-overexpressing HEK293T cells were cultured as described in the “HEK293T cell culture” section and seeded with a density of 5 × 10^3^ or 4 × 10^4^ cells per well in either 96-well or 24-well plates. The toxicity was assessed with the MTT assay (*61*). Following coincubation with artificial cells at given time points, the old growth medium was aspirated and replaced with non–FBS-containing fresh growth medium. After the addition of 10 μl of MTT per well in 200-μl final volume, the cells were incubated for 3.5 hours at 37°C. The growth medium was then replaced with 200 μl of DMSO to solubilize formazan salts. Following 15 min of incubation with shaking at 23°C, the absorbance was read at 590 nm, within 1 hour after adding DMSO. Triton X-100 [0.5% (v/v)] and 1 μM recombinant PFO were used as negative controls to assess complete cell death. The positive control was the no-treatment group. The percent cell viability was assessed by using these two boundaries.

### Immunoblotting

For experiments with cell-free extracts, in vitro transcription-translation reactions were diluted with 2× homemade Laemmli buffer and heated to 95°C for 10 min. An aliquot was loaded on 11% SDS–polyacrylamide gel electrophoresis (SDS-PAGE) and blotted using generic wet-transfer systems (300 mA, 2 hours). Nitrocellulose membranes (0.45 μm, Bio-Rad) were blocked using 5% (w/v) nonfat dry milk (Bio-Rad) in tris-buffered saline with Tween (TBST) containing 0.1% (v/v) Tween 20 and incubated with monoclonal primary antibody α-FLAG (M2) produced in mouse (Sigma-Aldrich, F3165) at either 23°C for 1 hour or 4°C for 16 hours with 1:5000 dilution in 5% milk in TBST. Following incubation with the primary antibody, membranes were washed three times with TBST and incubated 30 to 60 min at 23°C with horseradish peroxidase (HRP)–conjugated α-mouse polyclonal secondary antibody with 1:80,000 dilution in 5% milk in TBST. Then, the membrane was washed three times with TBST and the chemiluminescence signal was detected using ECL Select reagents (GE Healthcare) with a Bio-Rad ChemiDoc XRS+ and subsequently processed using ImageLab software (Bio-Rad). For the Western blotting of proteins from eukaryotic cells, the cell lysates were homogenized with multiple passages through a 25-gauge sterile needle and loaded onto SDS-PAGE (5 to 50 μl). The blotting and detection conditions were identical as above except that the nitrocellulose membranes were blocked with 2.5% (w/v) bovine serum albumin (BSA; Euroclone) or 3% milk in TBST. Experiments with eukaryotic cells used primary antibodies at the following dilutions: phospho-ERK1/2 with 1:2000 dilution in 2.5% BSA [Cell Signaling Technology (CST), no. 4370], total ERK1/2 with 1:2000 dilution in 2.5% milk in TBST (CST no. 9102), phospho-PLCγ1 with 1:2000 dilution in 2.5% BSA (CST no. 2821), total PLCγ1 with 1:2000 dilution in 2.5% milk/TBST (CST no. 2822), lamin A/C with 1:3200 dilution in 2.5% milk in TBST (Santa Cruz Biotechnology), calnexin with 1:2000 dilution in 2.5% milk in TBST (Santa Cruz Biotechnology), βIII-tubulin with 1:1000 dilution in 2.5% milk in TBST (Promega, G712A), and cleaved caspase-3 with 1:1000 dilution in 2.5% milk in TBST (CST, no. 9661s). The HRP-conjugated secondary antibodies were provided as follows: α-rabbit HRP conjugated with 1:2000 dilution in 2.5% BSA (Bio-Rad) and α-mouse HRP conjugated with 1:2000 dilution in 2.5% BSA (Bio-Rad).

### Immunofluorescence

All cells were fixed with 4% (w/v) paraformaldehyde after treatment. Upon 15- to 20-min incubation at room temperature, cells were washed three times with excess DPBS (without Ca^2+^ and Mg^2+^), subsequently permeabilized with 0.5% (v/v) Triton X-100 in PBS for 15 min at room temperature, and blocked with 5% (w/v) FBS and 0.3% (v/v) Triton X-100 in PBS for 2 hours at room temperature. Cultures were then incubated overnight at 4°C with specific primary antibodies (see below) diluted with 2% FBS and 0.2% Triton X-100 in PBS. The cells were then washed three times with PBS and incubated with the appropriate secondary antibodies described below for 2 hours at room temperature. Nuclei were counterstained with Hoechst 33258 (0.2 mg/ml) for 20 to 30 min at room temperature and washed three times with excess PBS before imaging. The primary antibodies were provided as follows: total TrkB with 1:1000 dilution (Santa Cruz Biotechnology, sc-377218), βIII-tubulin with 1:1000 dilution (Promega, G712A), cleaved caspase-3 with 1:500 dilution (CST, no. 9661s), MAP2 with 1:300 dilution (Chemicon-Millipore, AB5622), and glial fibrillary acidic protein with 1:1000 dilution (DAKO, Z0334). The secondary antibodies were provided as follows: α-rabbit Alexa Fluor 488 conjugated with 1:2000 dilution (Thermo Fisher Scientific) and α-mouse Alexa Fluor 633 conjugated with 1:2000 dilution (Thermo Fisher Scientific).

### Statistics

Unless otherwise indicated, all statistical analyses were performed with GraphPad Prism software version 7. For the experiments involving the release of GFP from artificial cells, the data from experiments in the presence and absence of 3OC6 HSL originated from the same batch of vesicles. Therefore, the statistical analysis exploited a paired *t* test instead of an unpaired *t* test. The cytosols were counted manually with Fiji (ImageJ), and the nuclei were counted either with Fiji (ImageJ) or with the Operetta instrument (PerkinElmer). The replicate numbers, population size, and statistical tests are provided in the figure legends. For the analysis of immunofluorescence and for the cell counting of GFP-expressing HEK293T cells, at least 3000 cells per condition for every antigen were counted. The immunostained and GFP-expressing cells were imaged with identical exposure times within the same experiment and exported from raw .czi files (Zeiss Zen software). Processing, histogram corrections, and quantification were with Fiji (ImageJ). The exposure times and histogram corrections are reported in table S3. Data were normalized by the total number of cells in every field, and the distribution was assessed for normality using the Shapiro-Wilk test. The statistical significance was determined by one-way analysis of variance (ANOVA) followed by a Dunnett’s post hoc test or two-tailed Student’s *t* test. A *P* value of less than 0.05 was considered statistically significant. All analyses were performed blind to the observer whenever possible.

## Supplementary Material

abb4920_SM.pdf

abb4920_Movie_S2.avi

abb4920_Movie_S1.avi

## SUPPLEMENTARY MATERIALS

Supplementary material for this article is available at http://advances.sciencemag.org/cgi/content/full/6/38/eabb4920/DC1

## Appendix

View/request a protocol for this paper from *Bio-protocol*.
