# Supplementary material for: Artificial cells drive neural differentiation
Source: Sci Adv. 2020 Sep 18;6(38):eabb4920. doi: 10.1126/sciadv.abb4920 (PMC7500934; doi:10.1126/sciadv.abb4920)
Supplement: abb4920_SM.pdf [file abb4920_SM.pdf]

Science Advances
AAAS

advances.sciencemag.org/cgi/content/full/6/38/eabb4920/DC1

Supplementary Materials for

Artificial cells drive neural differentiation

Ö. Duhan Toparlak, Jacopo Zasso, Simone Bridi, Mauro Dalla Serra, Paolo Macchi,
Luciano Conti, Marie-Laure Baudet, Sheref S. Mansy*

*Corresponding author. Email: sheref.mansy@ualberta.ca

Published 18 September 2020, *Sci. Adv.* **6**, eabb4920 (2020)
DOI: 10.1126/sciadv.abb4920

The PDF file includes:

Supplementary Text
Figs. S1 to S16
Tables S1 to S3
Legends for movies S1 and S2
References

Other Supplementary Material for this manuscript includes the following:

(available at advances.sciencemag.org/cgi/content/full/6/38/eabb4920/DC1)

Movies S1 and S2

Supplementary Text

Detailed figure legend description for Fig. 2.

Fig. 2. Artificial cells drive neuronal differentiation. (A) Overview of artificial cell ~~(AC)~~ treatment and mouse neural stem (mNS) cell differentiation strategy. ***Left***: Overview of signaling between artificial cells and neural stem cells. Homodimers of mature BDNF (green) act on the cognate receptor TrkB, activating signaling pathways through phosphorylation of $ERK1/2^{MAPK}$ (orange) via Shc (brown) and/or via PLCγ1 (purple). Both nodes lead to phosphorylation (red circle) and translocation of CREB and phosphorylated–CREB (p–CREB) into the nucleus, upon elevated cytosolic levels of cAMP and/or Ca^{2+}. This, in turn, activates the expression of genes behind a CRE–regulated promoter, which were pro–survival/pro–differentiation factors. ***Center***: Artificial cells were incubated with mNS cells for 15 days. To provide BDNF throughout differentiation and to avoid the effects of possible rupture (which was unlikely), Artificial cells were washed away and fresh artificial cells in fresh media were added every 24 h. The cartoon represents a cross–section of a cell culture well. ***Right***: After culturing mNS cells with Epidermal Growth Factor (EGF) and basic Fibroblast Growth Factor (bFGF) for 4 days, on day 0, EGF was removed and BDNF–secreting artificial cell levels were gradually increased starting with day 4, while bFGF was concomitantly decreased. **(B)** Representative immunostaining microscopy images of the differentiation of mNS cells into mature neurons *in vitro* at 19 days. Biologically inactive artificial cells (non–BDNF producing and secreting, i.e. sfGFP secreting, indicated as 'Background') were considered as a mock treatment and used for normalization (i.e. 0%) to assess the signal change between conditions with and without PFO pore synthesis (+/– 3OC6 HSL). **(C)** Statistical analysis of βIII–Tubulin overexpressing mNS cells. Cultures treated with commercial BDNF, i.e. Com. BDNF, were taken as a reference, i.e. 100% active represented by a dashed line. The data from panel B were used to generate the plot. Raw data before normalization are in fig. S4, F and G. **(D)** Representative western blot analysis of pre–differentiated mNS cells as a response to treatment with artificial cells at 19 days *in vitro*. Following 24 h of serum deprivation, pre-incubated artificial cells were co-incubated with the population of mNS cells for 30 min at 37 °C. For band intensity (densitometry) analysis, loading controls (Calnexin and Lamin A/C) and total PLCγ1 and total $ERK1/2^{MAPK}$ signals were used to normalize the intensities. Different membranes were used to accommodate antibody incompatibilities and the different sizes of protein. Scale bars indicate 50 μm, and data show ± SEM mean for n = 3 biological replicates, independent experiments. Statistical test was student's t–test (unpaired, two–tailed).

Supplementary Figures

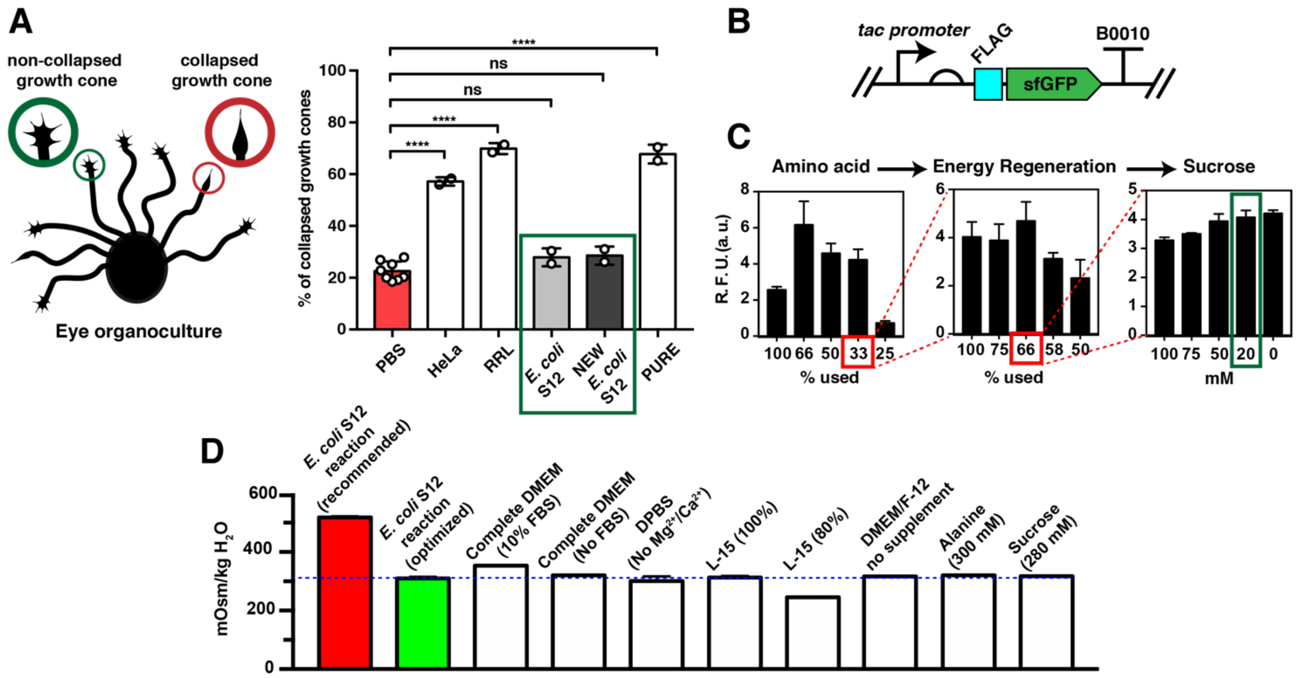

fig. S1. Cell–free reaction choice and optimization for physiological conditions. (A) Cytotoxicity of different cell–free extracts. *Left*: The assay, which is a highly sensitive *ex vivo* collapse assay, used cultured, non–dissociated neurons in the form of an organoculture to grow axons *de novo*. The health of the leading tip of the axon, i.e. the growth cone, was used as an indicator of toxicity. Unhealthy growth cones have a tendency to collapse when exposed to toxic compounds (determined as > 40% collapse). *Right*: The S12 *E. coli* extract was the least toxic to the axons, as bath application yielded physiological levels of collapsed growth cones. HeLa: HeLa cell lysate transcription translation reaction, RRL: rabbit reticulocyte lysate transcription–translation reaction, S12: *E. coli* S12 reaction, assembled from a homemade *E. coli* S12 extract, PURE: NEB PURExpress system. **(B)** Cartoon representation of the genetic construct (DT035A) used for the optimization of the reactions with the *E. coli* S12 extract. Sequence details are in the 'Genetic Constructs and Recombinant Proteins' section of the Methods. sfGFP denotes superfolder GFP. All reactions were at 30 °C for 12 h. **(C)** Optimization of the osmolality (to ca. 300 mOsm/kg H_2O) of the *E. coli* S12 reaction by evaluating the end–point expression levels of sfGFP from the genetic construct in panel (B). The difference in osmotic pressure between inside and outside of artificial cells would lead to the rupture of the vesicles; therefore, the osmolality of inner contents, i.e. the *E. coli* S12 reaction, was reduced to physiological levels. The reactions were assembled as reported in the Methods, *'in vitro transcription–translation'* section. Concentrated solutions of equimolar amino acid mixture and energy regeneration solutions contained the recommended values from the literature (*20*) and are reported in Table S1, along with the optimized amounts. The stock solution of sucrose was 500 mM and this solution was titrated down. Optimal amounts from each cycle is indicated by a red square and was used for the next round of optimization. Final conditions are indicated with a green rectangle. **(D)** Osmolality of the optimized *E. coli* S12 reaction compared to other solutions used in this study. For panel A, error bars represent ± SD of the mean for independent experiments (biological replicates) for n = 8 for PBS and n = 2 for the rest of the conditions. Statistical test was ordinary one–way ANOVA followed by Dunnett's multiple test, where **** is $p < 0.001$. For panel (C), error bars represent ± SD of the mean from independent experiments with n = 3.

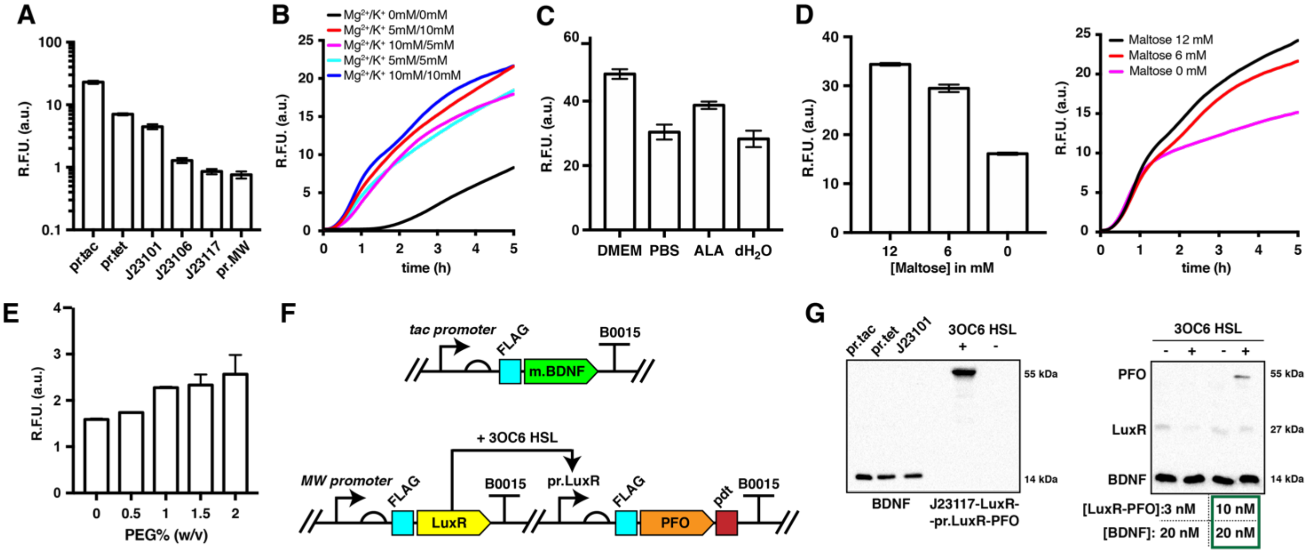

fig. S2. Details of the optimization of the *E. coli* S12 reaction for physiological conditions. *In vitro* transcription–translation reactions were at 30 °C for 12 h. Bar graphs show final time points. Fluorescence experiments monitored the emission arising from the expression of superfolder GFP from construct DT035A (same as fig. S1) **(A)** The effect of different transcriptional promoter strengths. All N–terminal sequences were identical except for the promoter region. Details on the promoters are in the Methods and Table S1. **(B)** Effect of supplementary solutions on the synthesis of sfGFP, Mg^{2+}–glutamate/K^{+}–glutamate denoted as Mg^{2+}/K^{+}. **(C)** Effect of potential contaminants of the optimized S12 reaction. PBS, DMEM, alanine (300 mM in nuclease–free water) were added as 10% (v/v) of the reaction volume. No negative effect was detected compared to the positive control, which contained nuclease–free deionized water. **(D)** *Left*: Effect of maltose on the end point (10 h). *Right*: Effect of maltose on early transcription–translation. **(E)** Effect of the macromolecular crowding agent PEG 8000 on transcription–translation. Values above 2% (w/v) PEG 8000 gave higher yield; however, due to the increased osmolality of the *E. coli* S12 reaction, we chose 2% (w/v) for further experiments. **(F)** Cartoon representations of each construct. m.BDNF indicates mature BDNF (DT033A), pr.MW is a hybrid transcriptional promoter (BBa_J23106 and BBa_J23117, DT034A), and pdt is a *Mesoplasma florum* protein degradation tag (*62*). **(G)** *Left*: Western blot analysis of BDNF, LuxR, and PFO expression for the optimization of plasmid concentration. Immunoblotting was against the N–terminal FLAG–tag. Strong transcriptional promoters were screened for BDNF. Hybrid weak promoter of LuxR allowed for the synthesis of PFO by *in vitro* transcription–translation upon induction with 10 μM 3OC6 HSL. To ensure that the limited resources of the artificial cell were primarily directed toward the synthesis of BDNF (*63*), strong and weak promoters were selected for the expression of BDNF and LuxR, respectively. *Right*: Optimal plasmid concentrations (green) for protein production were 20 nM for the BDNF plasmid (DT033A) and 10 nM for the LuxR–PFO plasmid (DT034A). Induction of PFO expression was after 45 min incubation at 30 °C.

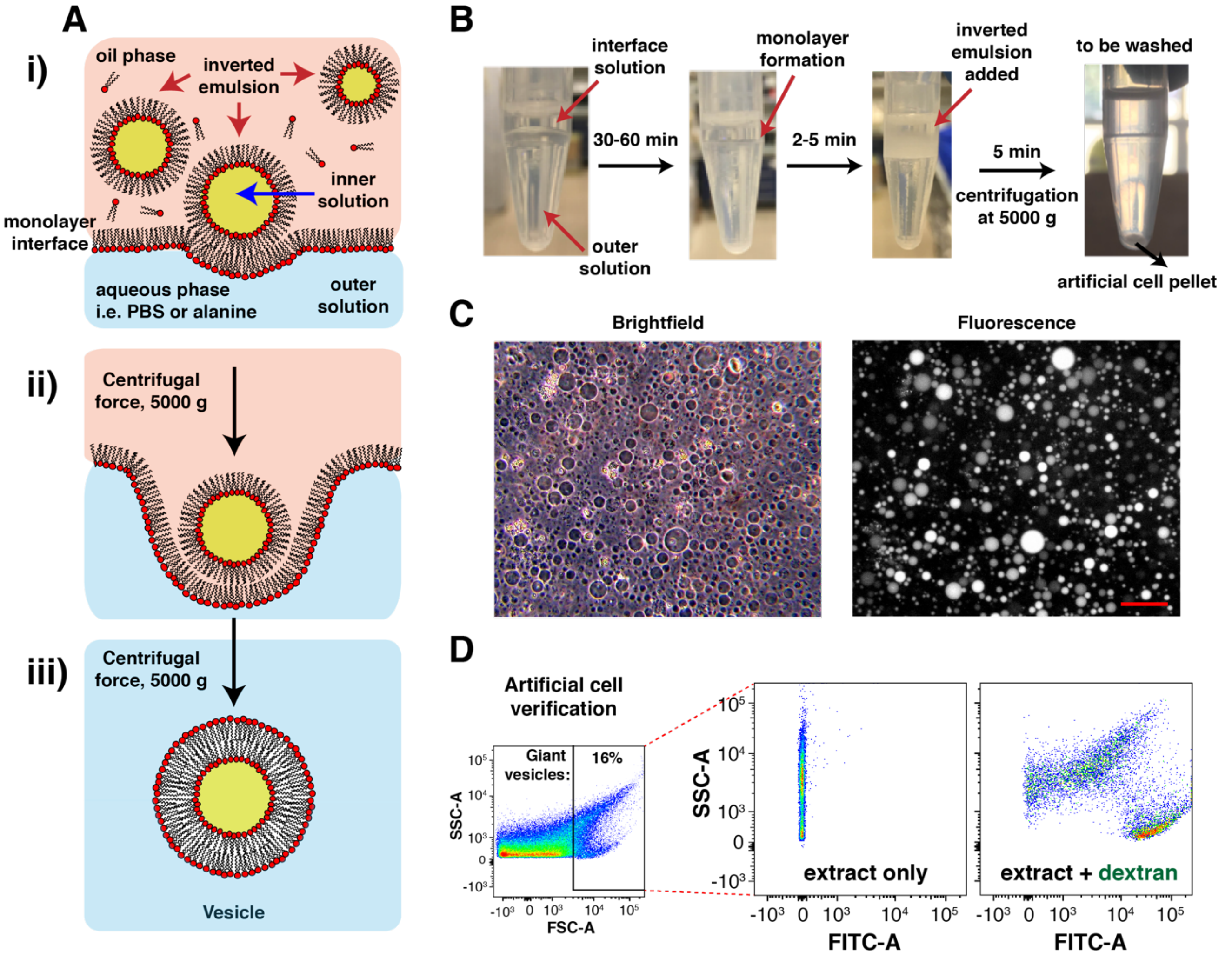

fig. S3. Overview and validation of vesicle formation by the inverted emulsion method. (A) A mineral oil (light oil, ρ = 0.84 g/mL) solution was prepared to dissolve the lipid components. Water–in–oil (inverted) emulsions were prepared with 2% (v/v) aqueous solution in the lipid–mineral oil phase. For example, 10 µL of inner solution was pipetted into 500 µL lipid/mineral oil solution. Subsequently, the 1.5 mL tube containing the mixture was rapidly drawn over a rack to form inverted emulsions (see Methods for more details). A mild centrifugal force was applied to form vesicles taking advantage of the density difference between outer ($\rho_{alanine}$ = 1.42 g/mL) and inner solutions ($\rho_{sucrose}$ = 1.59 g/mL). **(B)** Pictures illustrating the process of forming vesicles. Photo Credit: Ömer Duhan Toparlak, University of Trento. **(C)** Microscopy images of sucrose containing vesicles generated by the inverted emulsion technique described in panel (A). The inner solution composition was 280 mM sucrose and 500 µM HPTS. An equiosmolar alanine solution (i.e. 300 mM) or DPBS (without Mg^{2+} and Ca^{2+}) was used as outer solution, to take advantage of density difference between inside and outside the vesicles. Identical results were obtained with the *E. coli* S12 extract. To enhance the density difference and the efficiency of the cell–free reaction, the inner solution was further supplemented with 20 mM of sucrose and 6 mM of maltose (fig. S1C and S2D). Scale bar indicates 100 µm. **(D)** Flow cytometry analysis of artificial cells generated by inverted emulsion technique. The inner solution for artificial cells contained osmotically optimized *E. coli* S12 reaction and 10 µM AlexaFluor488 labeled 10 kDa dextran. The instrument was calibrated with polystyrene beads of known size. Upon calibration, the giant vesicle fraction was selected for uniform particle distribution and reliable characterization of the artificial cell population.

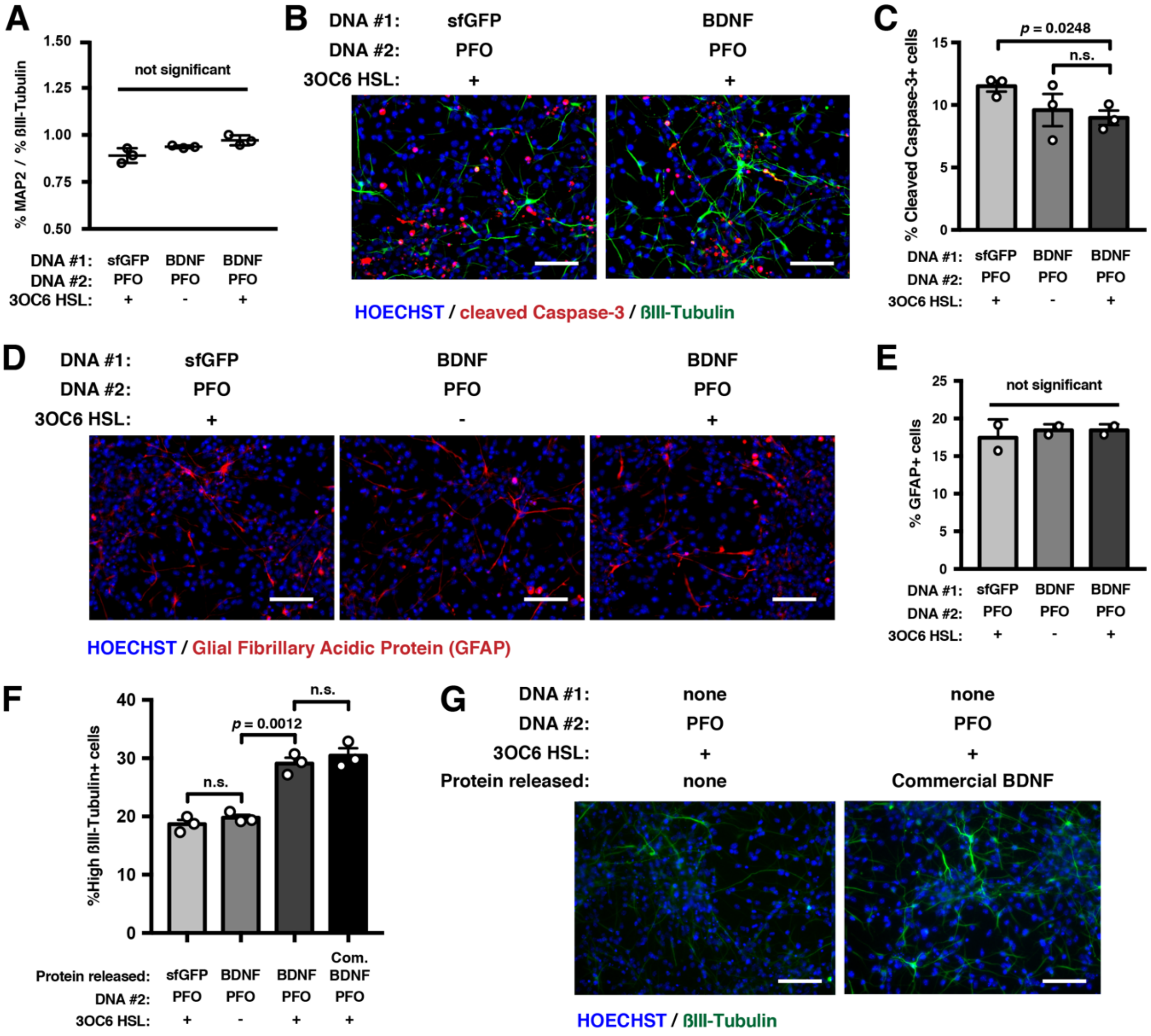

fig. S4. Supporting data for artificial cell functionality with neural stem cells. (A) Statistical analysis of MAP2 and βIII–Tubulin immunostaining. Overlapping signals were used to assess the maturation levels and signal specificity. Data indicate the mean of three different fields with ± SD. The statistical test was student's t–test (unpaired, two–tailed), where p < 0.05 was considered significant. **(B)** Representative microscopy images of immunostaining of mNS cells at 19 days *in vitro*, after artificial cell treatment, antibodies were against cleaved Caspase–3 (red) to assess apoptosis and βIII–Tubulin (green) to assess the success of differentiation to appreciate non–overlapping cleaved Caspase–3 signals. **(C)** Statistical analysis of overall cell population for cleaved Caspase–3. Data indicate mean of three independent experiments (n = 3) and error bars indicate ± SEM. The statistical test was student's t–test (unpaired, two–tailed), where p < 0.05 was considered significant. 'n.s.' stands for 'not significant'. **(D)** Representative immunostaining images of mNS cells at 19 days *in vitro*, after artificial cell treatment. α–Glial Fibrillary Acidic Protein (GFAP, red) antibody was used to assess the glial cells i.e. astrocyte population during differentiation and maturation with artificial cells. **(E)** Statistical analysis of overall cell population for GFAP. Data indicate the mean of two different fields with ± SD. The statistical test was student's t–test (unpaired, two–tailed), where p < 0.05 was considered significant. **(F)** Raw data for the differentiation of mNS cells into neurons. An artificial cell formula encapsulating commercial BDNF and releasing upon sensing 3OC6 HSL was used to assess the quality of neural differentiation. "Com. BDNF" stands for Commercial BDNF (Peprotech) All data points are biological replicates. This dataset was used to generate Fig. 2C. **(G)** The representative microscopy images of no–protein and commercial BDNF–encapsulating and secreting artificial cells at 19 days *in vitro*. All scale bars indicate 50 μm.

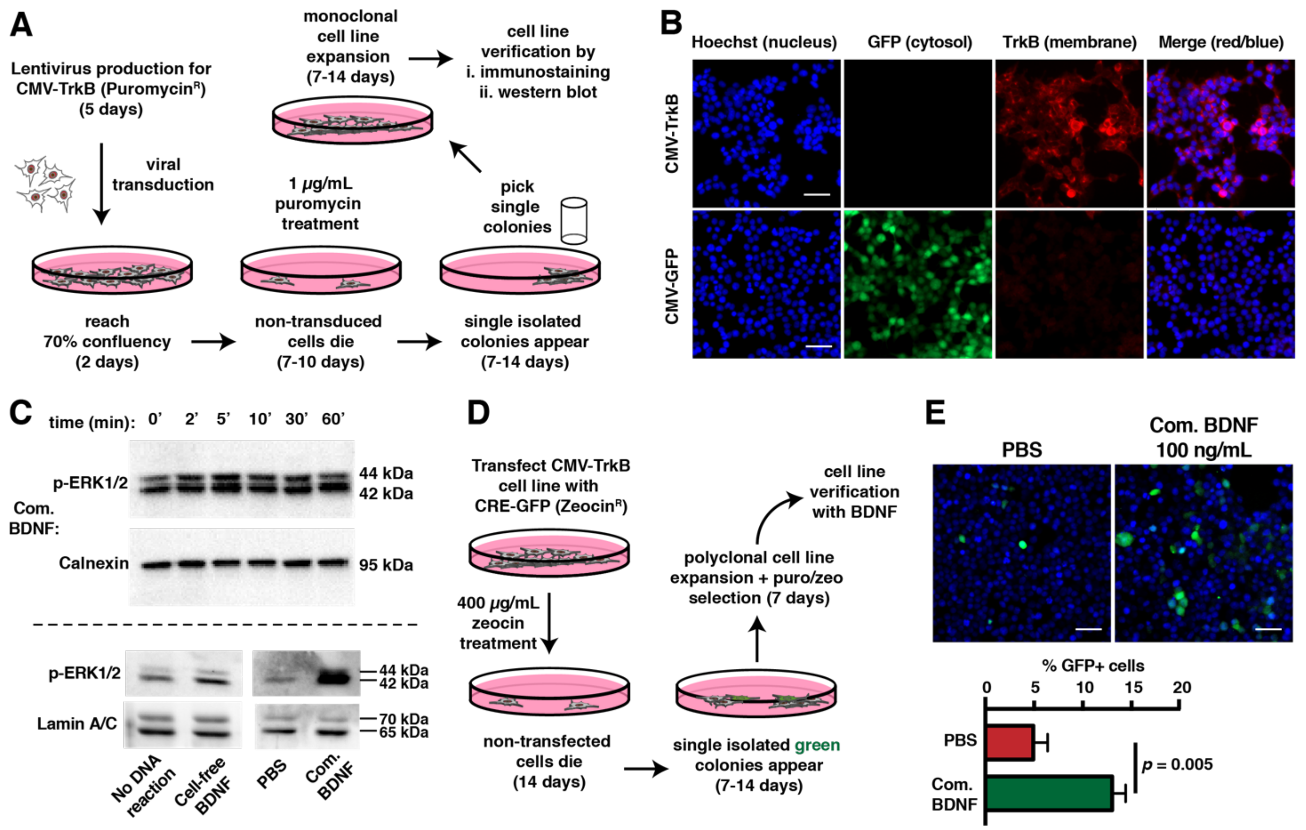

fig. S5. Validation of the Human Embryonic Kidney 293T (HEK293T) cell lines. (A) Outline of the procedure for the generation of the CMV–TrkB monoclonal cell line, where the gene expression of Tropomyosin Kinase B (TrkB) was controlled by Cytomegalovirus (CMV) enhancer and promoter regions. **(B)** Immunofluorescence staining of the low–TrkB–overexpressing cell line, where CMV–GFP transduction was used as a background signal for α–TrkB immunostaining. Approximately 15 different clones were screened, and the lowest TrkB expressing cell line was selected to reduce the potential noise arising from the autophosphorylation of the overexpressed TrkB receptor. **(C)** Western blot analysis of the activation of the TrkB pathway in the HEK293T–CMV–TrkB cell line. *Upper*: Time–based detection of phosphorylated–ERK1/2^{MAPK} (both isoforms, 42 and 44 kDa) using Calnexin signals as SDS–PAGE loading control. "Com. BDNF" stands for commercial BDNF, which was used at a final concentration of 20 ng/mL. *Lower*: Activation of the TrkB–BDNF signaling pathway by commercial BDNF (20 ng/mL) at 10 min. The band intensity analysis showed a 400% difference in phosphorylated–ERK1/2^{MAPK} when compared to PBS. **(D)** Generation and validation of the TrkB/CRE–GFP polyclonal cell line starting from the CMV–TrkB monoclonal cell line. **(E)** Response of the CRE–GFP cell line to commercial BDNF. Commercial BDNF was used at a final concentration of 100 ng/mL. Western blotting was performed for n = 2 independent experiments (biological replicate), and cell counting was performed for n = 3 independent experiments (biological replicate). The statistical tests were student's unpaired t–test where p value less than 0.05 was considered statistically significant. All scale bars indicate 50 μm.

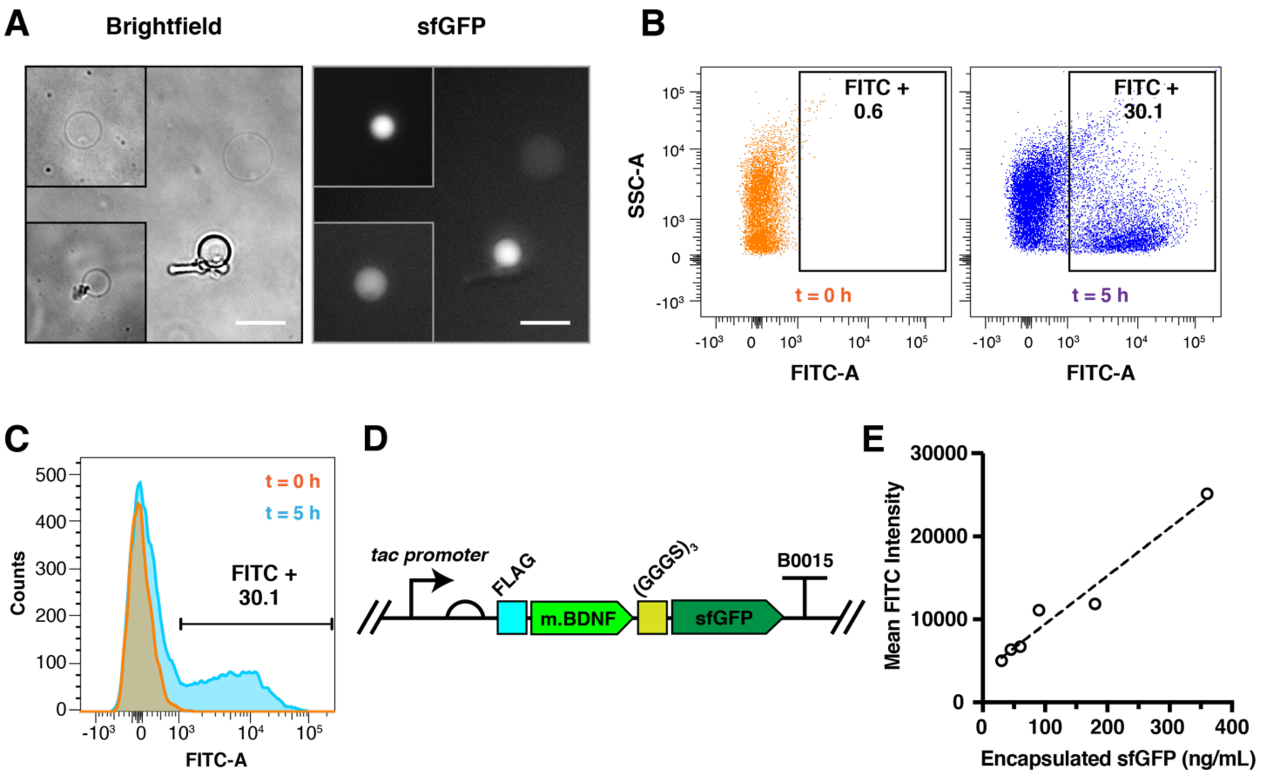

fig. S6. Characterization of protein production inside of artificial cells. (A) Microscopy images of sfGFP producing artificial cells. Scale bars indicate 10 μm. **(B)** Flow cytometry analysis of artificial cells that synthesize sfGFP. The dot plot is of data at t = 0 and t = 5 h. **(C)** Histogram overlap for t = 0 and t = 5 h. The reaction was at 30 °C. **(D)** Chimeric protein construction for N–terminal FLAG–tagged BDNF–sfGFP. **(E)** Standard curve for the determination of the concentration of BDNF–sfGFP inside of artificial cells. Artificial cells contained known amounts of 6xHis–sfGFP with optimized extract composition. After the gating strategy was applied to select for giant vesicles (fig. S3D), the mean FITC intensities were calculated for known 6xHis–sfGFP concentrations and used to determine the sfGFP synthesized inside of the artificial cells. The average sfGFP concentrations were then extrapolated from the linear fit of the standard curve.

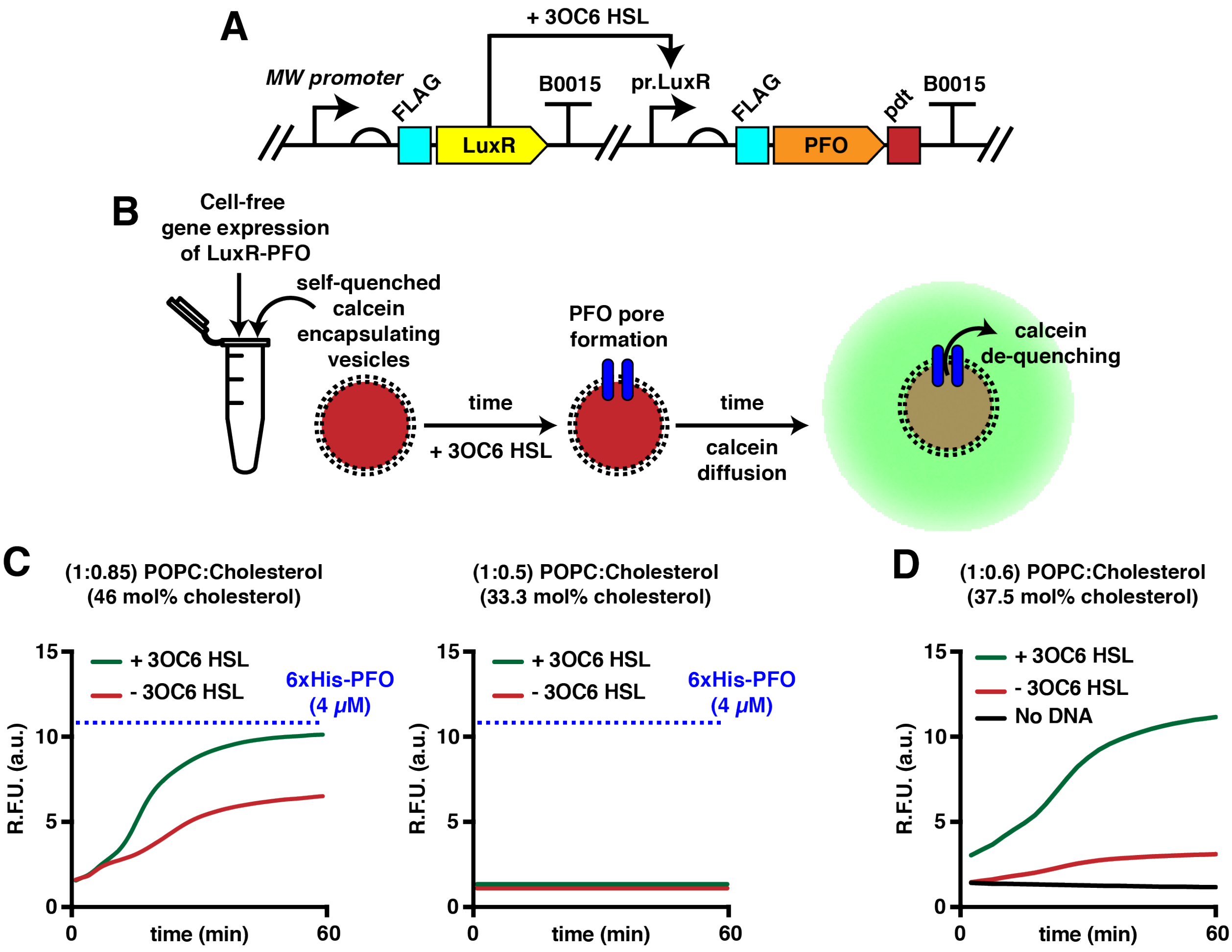

fig. S7. Choice of membrane composition and PFO functionality test. (A) Cartoon representation of the genetic circuit used. **(B)** Calcein leakage assay was used to assess the functionality of PFO and to screen cholesterol concentrations. POPC:cholesterol vesicles were prepared by the hydration of Freeze–Dried Empty Liposomes (FDEL) (*51, 52*). 100 nm diameter vesicles containing 80 mM calcein were added to *in vitro* transcription–translation reactions at 30 °C and the change in fluorescence was monitored over time. Upon the induction of synthesis of PFO with 10 μM 3OC6 HSL, an increase of fluorescence was observed, indicating the synthesis and assembly of functional pores. Increased fluorescence was due to the dequenching of fluorescence in more dilute solutions external to the vesicles. **(C)** Selection of the artificial cell membrane composition for functional PFO pores and vesicle formation. Optimized plasmid concentrations (fig. S5) were used for the expression of PFO (DT034A). 1:0.5 POPC:cholesterol membranes did not allow for the formation of a functional PFO pore. Dashed lines indicate the maximum fluorescence value achieved either by adding 0.5% (v/v) Triton X–100 or 4 μM 6xHis–tagged PFO that was recombinantly expressed and purified. **(D)** Influence of a C–terminal small protein degradation tag (pdt) (see also fig. S3 and Table S2) on the efficiency of PFO pore formation was investigated. No significant inhibition or loss of function was detected. While 1:0.6 POPC:cholesterol membranes also allowed for the formation of PFO pores, 1:0.85 POPC:cholesterol was chosen for subsequent experiments to compensate for potential changes in the composition of the membrane due to the exploitation of the inverted emulsion technique.

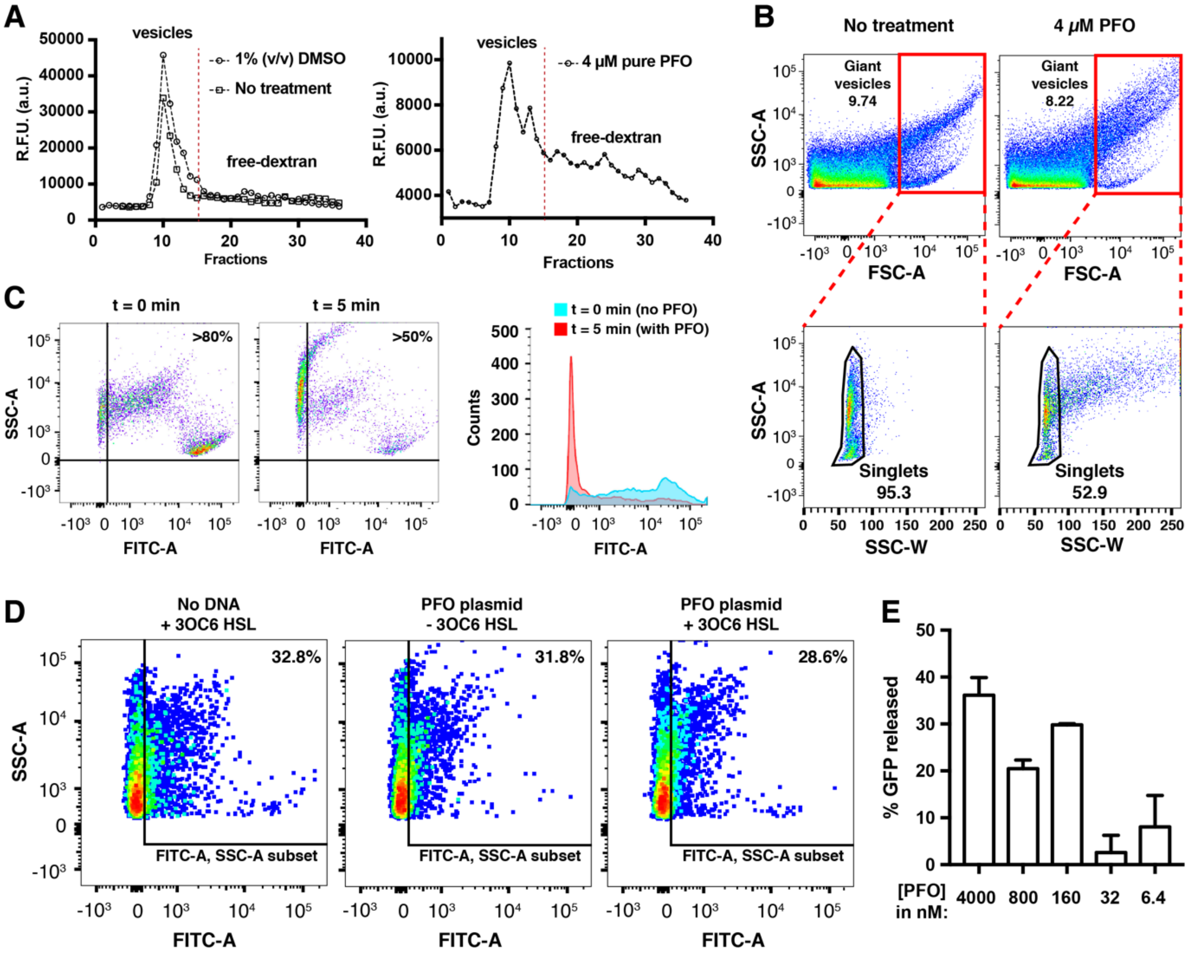

fig. S8. Analysis of vesicles containing sucrose and *E. coli* S12 reaction. (A) Characterization of content leakage induced by recombinantly expressed and purified PFO. Vesicles contained 280 mM sucrose in PBS and 10 μM AlexaFluor488 labelled 10 kDa dextran. Vesicles were prepared as indicated in fig. S6, purified and washed with excess PBS. Loss of entrapped material was assessed by size–exclusion chromatography with a sepharose 4b column, as previously described (*60*). Vesicles eluted between fractions 9–15. Leaked material eluted after fraction 15, indicated with the red dashed line. The 3OC6 HSL stock solution was stored in 100% DMSO and diluted in water prior to the induction of gene expression, giving a final concentration of 1% (v/v) DMSO. Therefore, the negative control contained 1% (v/v) DMSO in the vesicle solution. **(B)** Flow cytometry analysis of PFO–membrane association. Vesicles were prepared as indicated in panel (A). Single events decrease, and multiple events increase with PFO–membrane association, presumably due to vesicle–vesicle interactions triggered by PFO oligomerization. **(C)** Flow cytometry demonstration of PFO–induced content release from *E. coli* S12 reaction encapsulating artificial cells. All artificial cells contained 10 μM 10 kDa AlexaFluor488–labelled dextran and no DNA template. *Left*: Flow cytometry analysis after 5 min of PFO exposure, dot plot analysis. *Right*: Histogram overlap of t = 0 and t = 5 min data. **(D)** Representative flow cytometry data demonstrating the functionality of the PFO pores and release of encapsulated GFP as described in Fig. 4 and the Methods section. Artificial cells were prepared as in '*Generation of artificial cells*' section in the Methods and contained plasmid DNA encoding LuxR and PFO, under weak and 3OC6 HSL inducible transcriptional promoters, respectively. No DNA encapsulating GFP–containing vesicles were taken as 100% reference (Leftmost). Here DNA template containing vesicles gave ca. 3% and ca. 13% release of GFP, without and with 3OC6 HSL, respectively. **(E)** Titration of recombinant PFO with artificial cells in bulk solution. Release of GFP was monitored by FACS. Error bars are ± SD. All incubations were at 30 °C for 16 h.

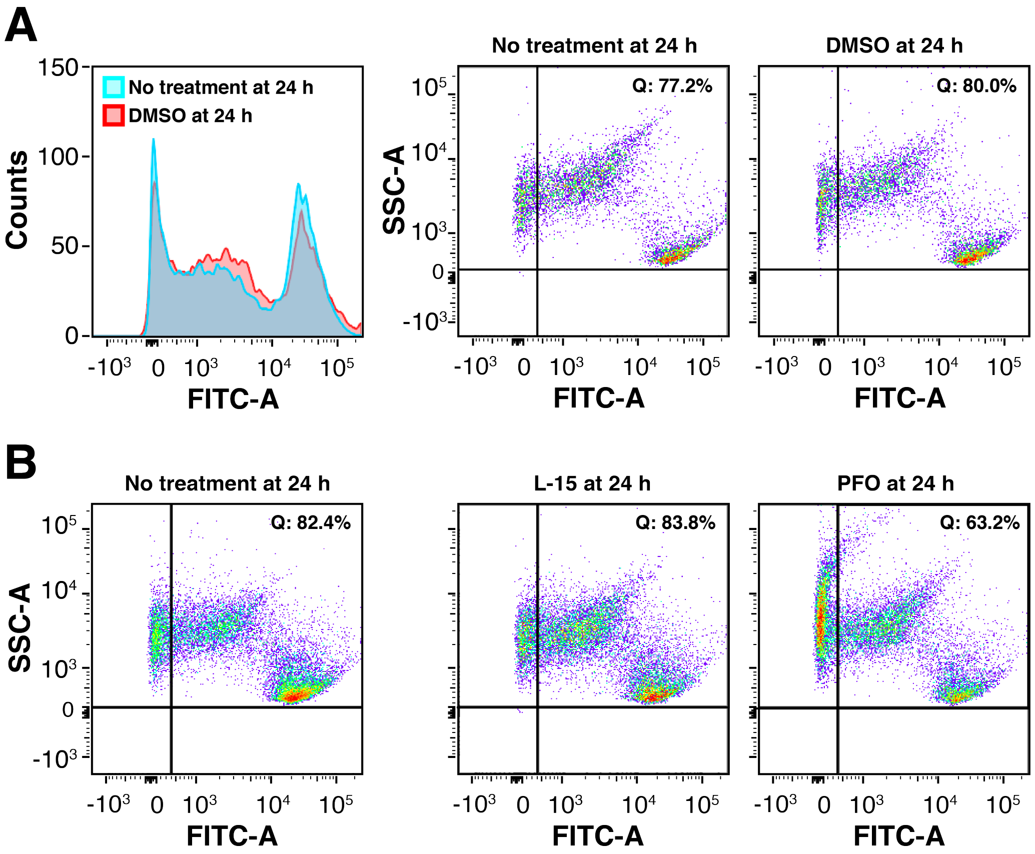

fig. S9. Stability of artificial cells under physiological conditions at 24 h. All artificial cells contained *E. coli* S12 reaction, 10 μM 10 kDa AlexaFluor488–labelled dextran and DNA encoding LuxR and PFO (DT034A) and were incubated at 30 °C without induction. Unless otherwise noted, all artificial cells were incubated in PBS. **(A)** Effect of 1% (v/v) DMSO on the stability of artificial cells. *Left:* A histogram plot comparison of the artificial cells "No treatment at 24 h" and "DMSO at 24 h". *Right:* Dot plot analyses of the histogram data, "No treatment at 24 h" quadrant was 77.2% and the "DMSO at 24 h" quadrant was 80%. No significant difference was observed. Vesicles were obtained from identical batch of preparations. **(B)** Flow cytometry analysis of the stability of the artificial cells upon treatment with 80% L–15 media (osmolality reduction of 20% from PBS) as "L–15 at 24 h" and 4 μM PFO, as "PFO at 24 h". Quadrants were as following, from left to right, "No treatment at 24 h" was 82.4%, "L–15 at 24 h" was 83.8% and "PFO at 24 h" was 63.2%. Vesicles in panel (B) were from a different batch of preparation panel (A).

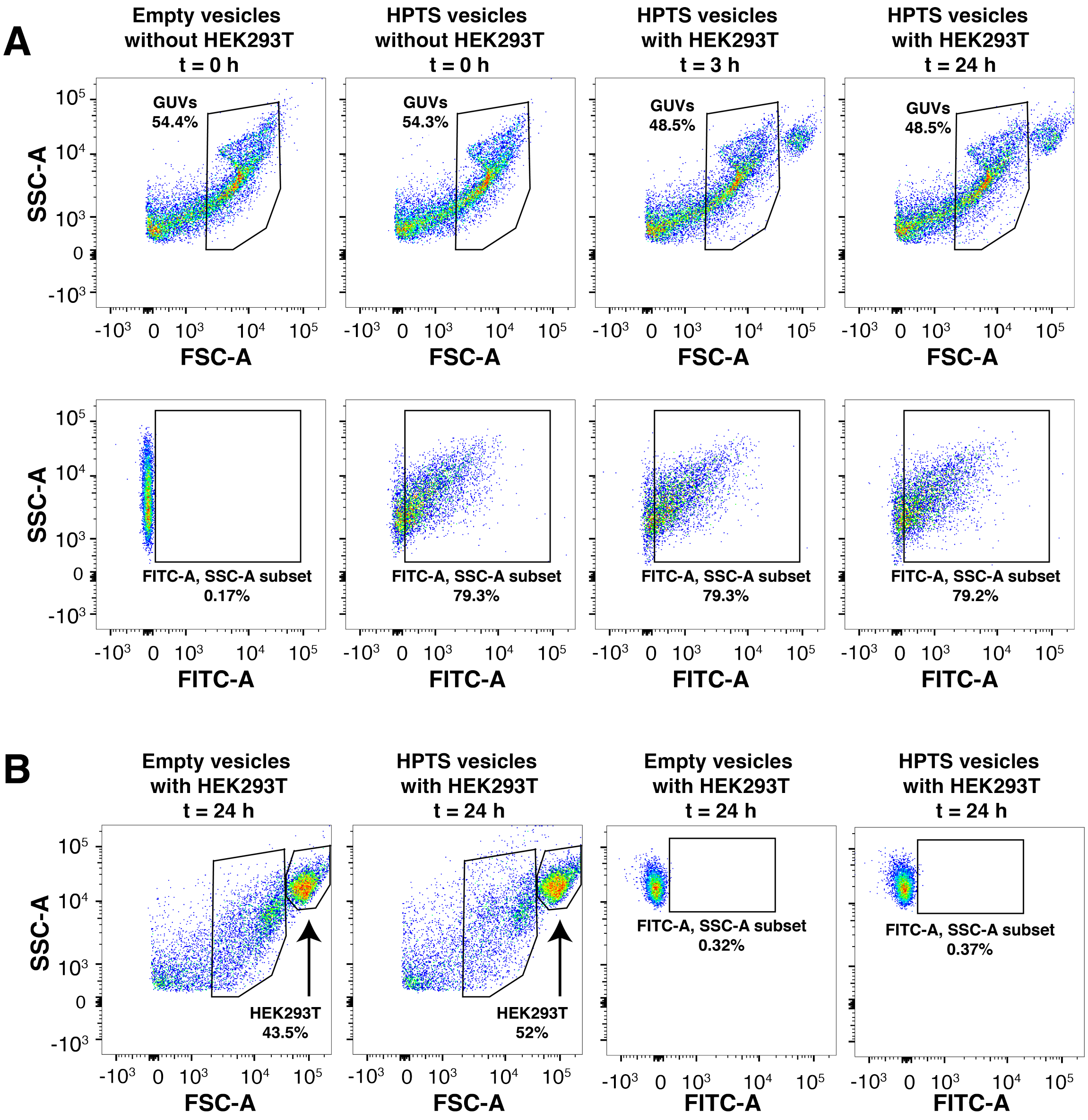

fig. S10. Stability of artificial cells in the presence of eukaryotic cells at 24 h. For the side–scatter (SSC–A) vs forward–scatter (FSC–A) plots, a gating strategy was applied to differentiate artificial cells from eukaryotic cells (upper row). Subsequently (lower row), the FITC channel was used to determine the fluorescence of the artificial cells over time. **(A)** Analysis of vesicle rupture. Pyranine (HPTS)– and S12–encapsulating (without DNA) artificial cells were co-incubated with HEK293T cells for 24 h at 37 °C. Prior to analysis by flow cytometry, cells (together with artificial cells) were detached from the culture dish by trypsinization, diluted in DMEM, and analyzed without fixation. The data from different time points indicated that no significant vesicle rupture was present after 24 h of co–incubation, as can be assessed by the unchanged percentage of vesicle fluorescence. No significant drop in fluorescence was detected after 24 h of co-incubation. **(B)** Analysis of potential vesicle–eukaryotic cell association. Artificial cells of the same formulation as in panel (A) were then washed away and the eukaryotic cell population was analyzed for an increase in HPTS fluorescence. *Left*: The same gating strategy as in panel (A) was applied to exclude artificial cells. *Right*: the FITC channel was used to determine the fluorescence intensity of eukaryotic cells, which might have possibly increased due to fusion events. No significant fluorescence increase was detected (0.05% change, within reasonable handling and background error). Data were reproduced at least twice on different days.

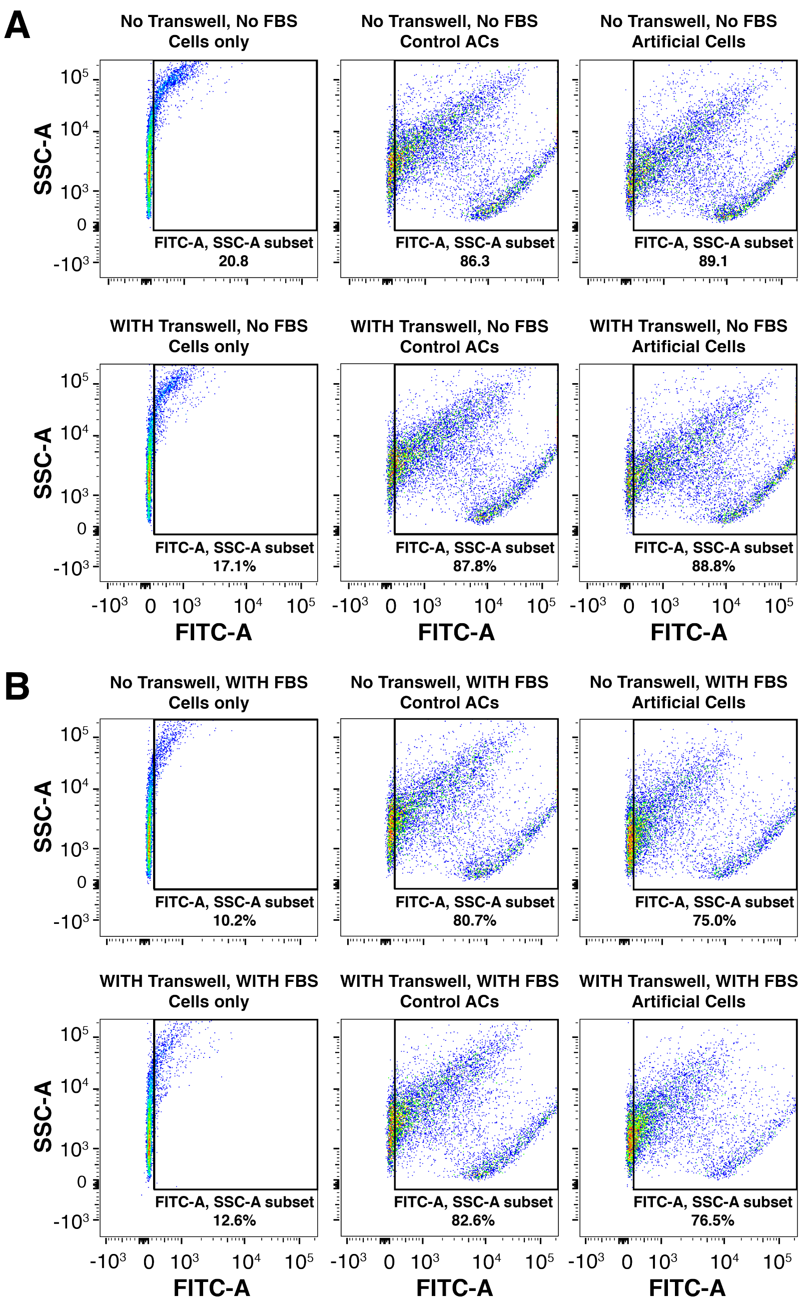

fig. S11. Stability of artificial cells under eukaryotic cell growth conditions. All artificial cells encapsulated S12 reaction with pyranine (HPTS) and all co-incubations were at 37 °C. **(A)** Stability of artificial cells without FBS at 24 h. The HEK293T cells and artificial cells were incubated either with or without the use of a Transwell, as depicted in the assays to simulate experimental conditions in Fig. 2 and Fig. 3. **(B)** Stability of artificial cells with 10% (v/v) FBS at 24 h. Artificial cells that were not in contact with eukaryotic cells are denoted as a control group and used as a reference for maximum FITC+ events, in both FBS and Transwell tests. In this group, the artificial cells were separately incubated at 37 °C in PBS for 24 h and then briefly washed with eukaryotic cells and isolated immediately, in order to match the same culture environment as in test group. In the presence of FBS, the population of artificial cells showed a ca. 7% decrease within 24 h, taking the control vesicles group as 100%. However, this effect was not observed in the absence of FBS. Presumably the rapid increase in osmolality, unknown components of the bovine serum, and an increase in eukaryotic cell-derived events due to cell growth are the three contributing factors for the loss of artificial cell events.

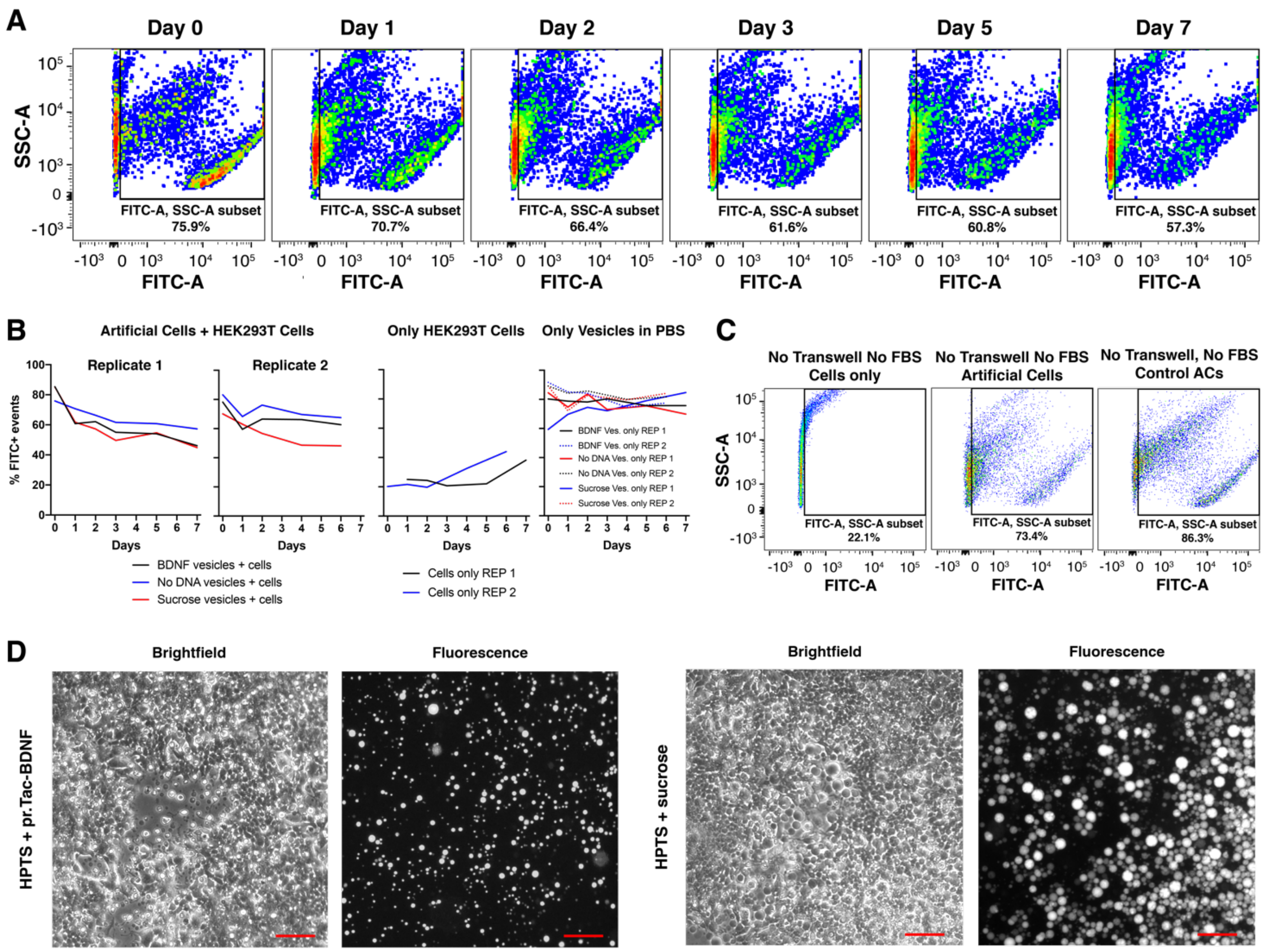

fig. S12. Stability of artificial cells and the growth of HEK293T cells over one week. All artificial cells encapsulated S12 reaction with pyranine (HPTS) and all co-incubations were at 37 °C. **(A)** Representative flow cytometry results, over the course of one week of artificial cell and HEK293T cell co-incubation and cellular growth in the presence of 10% (v/v) FBS in complete DMEM at 37 °C. **(B)** Artificial cell death–curve over the course of one week at 37 °C. *Left*: Artificial cell events showed up to a 30% decrease in the presence of FBS. The decreasing trend of artificial cell events did not change significantly in the presence or absence of plasmid DNA encoding BDNF. While FITC+ artificial cell events decreased, the FITC+ events of eukaryotic cellular origin increased ("Only HEK293T Cells"). The influence of cellular debris and extracellular vesicles to the fluorescent events were found to be present in up to 10–20% of the significant events. Whereas, the vesicles incubated in PBS showed an overall 5–10% decrease, indicating that the loss of artificial cells was not primarily due to the eukaryotic cells. **(C)** The stability of the artificial cells in the absence of FBS after one week at 37 °C. Taking into account the control vesicle group (as in fig. S11), the artificial cell events decreased only by 15% after one week of co-incubation. **(D)** Microscopy images to determine the compatibility of artificial cells with HEK293T cells after one week of co-incubation at 37 °C. The HEK293T cells were grown in FBS-containing media. Overly confluent HEK293T cells overlap and integrate with artificial cells. The presence of artificial cells did not disturb the growth of eukaryotic cells. Further, overly confluent eukaryotic cells did not induce the lysis or rupture of the vesicles, either by chemical or mechanical means. All scale bars indicate 100 µm.

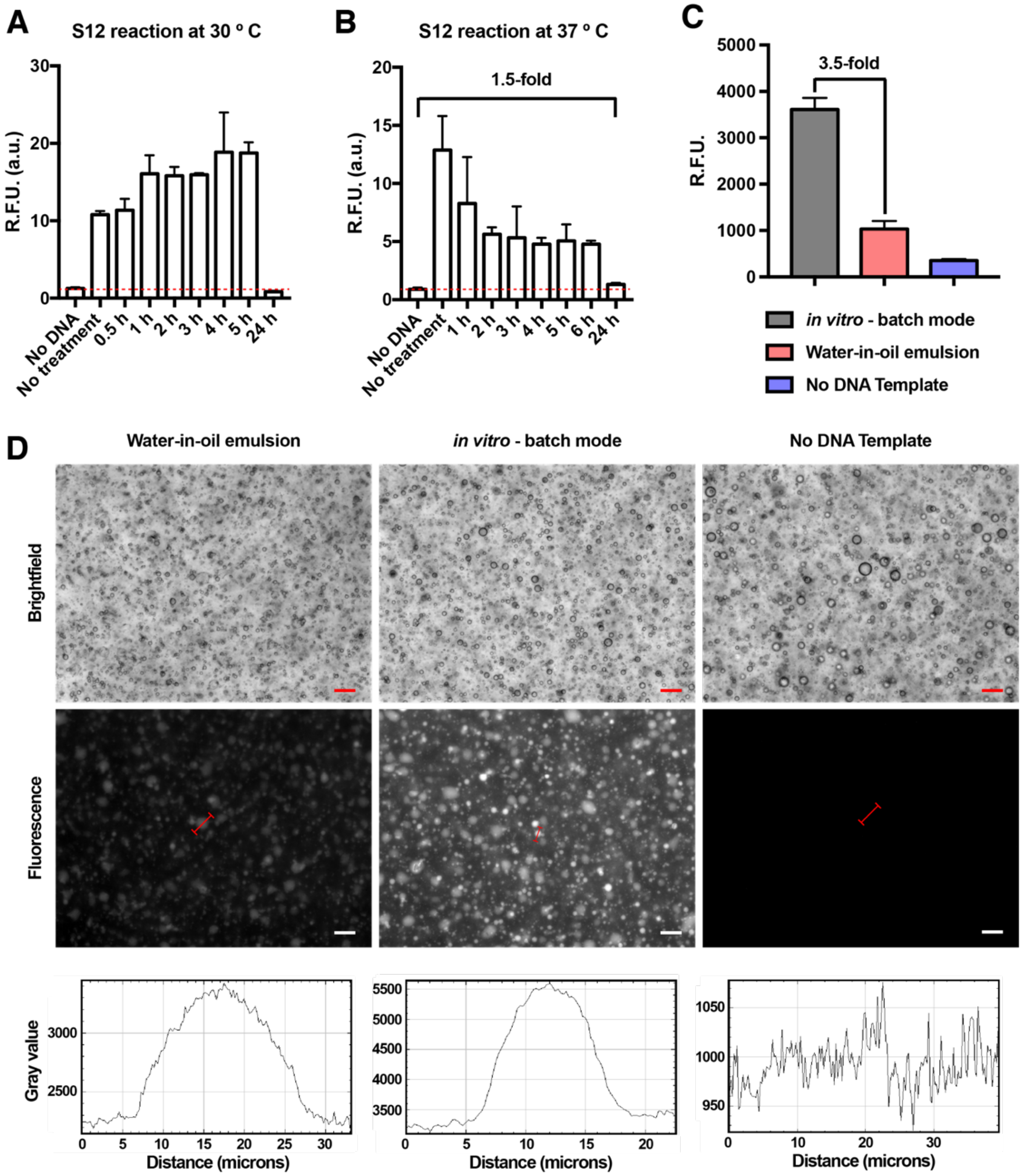

fig. S13. Activity of the S12 extract under different conditions. (A) Cell–free extract stability at 30 °C. (**B**) Cell–free extract stability at 37 °C. 1.5–fold signal difference –with respect to the no DNA reaction– was detected after 24 h of incubation before addition of template DNA encoding sfGFP. This value corresponds to the batch production of ca. 16 µg/mL sfGFP (see fig. S16 for calculations) **(C)** Comparison of *in vitro* (batch mode) with water–in–oil emulsion sfGFP production. For batch mode, the reactions were first performed in 0.2 mL PCR tubes, and afterwards encapsulated inside the emulsions, to reach the identical environment. The readings were from Tecan M200 plate reader following 16 h of incubation at 30 °C. **(D)** Representative examples of emulsions generated and analyzed. *Upper*: Microscopy images of sfGFP intensity inside emulsions. *Lower*: Example line-plot-profile for a representative emulsion signal intensity. For all experiments, the template DNA was 20 nM DT035A. For (A) and (B), the template DNA was added to the reaction mixture at indicated time points, following incubation without DNA at indicated temperatures. All scale bars are 20 µm.

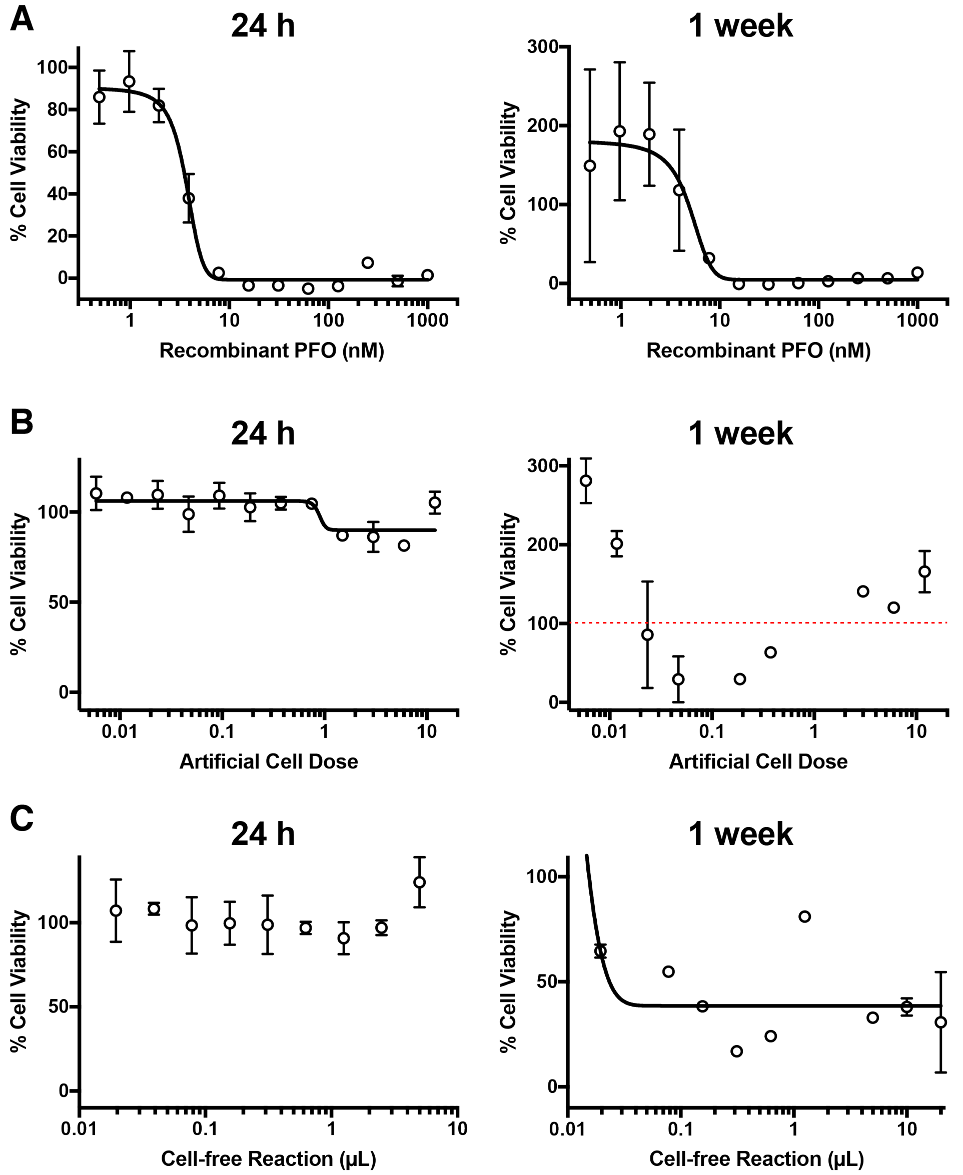

fig. S14. Toxicity of artificial cells to HEK293T cells. **(A)** MTT assay results following co-incubation with recombinantly expressed and purified PFO. Inhibitory concentration (IC_{50}) of recombinant PFO was found to be ca. 3.7 nM for HEK293T cells. This value is likely to be a higher concentration of PFO than artificial cells can produce and release. This control was used as a reference. These data combined with the data in fig. S8E were consistent with intravesicularly produced and/or leaked amounts of PFO roughly equal to or less than 4 nM. **(B)** MTT assay results following co-incubation with no-DNA-containing artificial cells at days 1 and 7. No significant cytotoxic effects were observed with HEK293T cells. The erratic readings are due to cell growth and seeding differentials, as similar growth deficiencies were also observed in non–treated wells. A single dose of artificial cells is determined as the final amount used to generate Fig. 2. **(C)** MTT assay results following co-incubation with no–DNA-containing cell–free extract at days 1 and 7. Within 24 h, the cell–free reaction dose used with HEK293T cells for the assessment of the functionality of cell–free synthesized BDNF (0.25 μL) did not show any cytotoxic effect. Following 1-week incubation, the cell–free reaction began to exert a cytotoxic effect. In these assays, the complete cell death was achieved by adding Triton X–100 at a final concentration of 0.5 % (v/v), which was the negative control. The positive control was the no–treatment group. The positive control was considered 100% cell viability, whereas the negative control was used as 0% cell viability. The data were fitted to sigmoidal, where X values were considered as concentration, to generate IC_{50} values on GraphPad Prism 7.

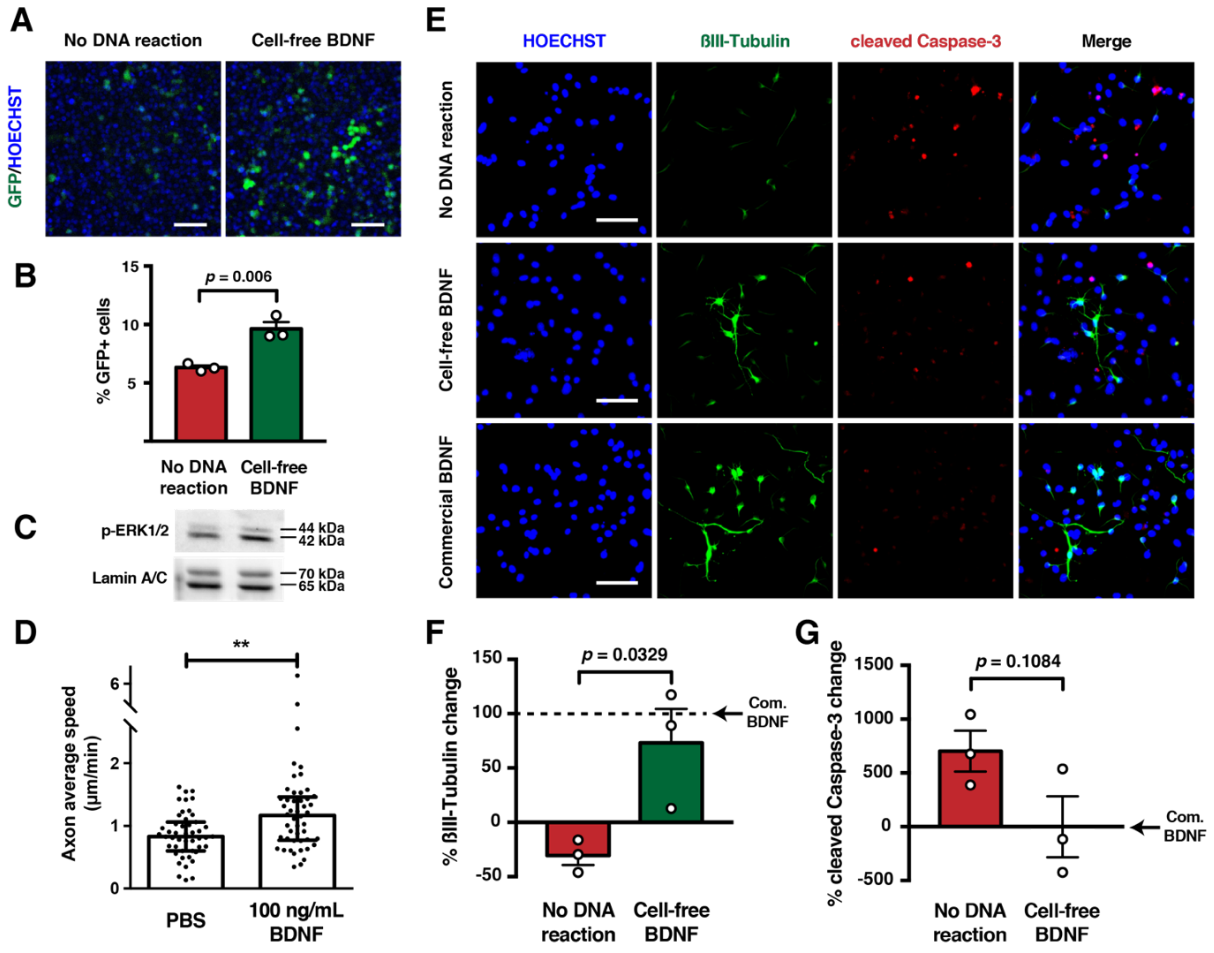

fig. S15. Characterization of cell–free synthesized BDNF. (A–C) with engineered HEK293T cells, **(D)** with *Xenopus laevis* RGC axons, **(E–G)** with neural stem cells. **(A)** Representative expression of GFP in genetically engineered HEK293T cells treated with cell–free expressed BDNF. Scale bars indicate 100 µm. **(B)** Statistical analysis of GFP positive HEK293T cells represented in panel a. Data show the mean. The error bars represent ± SEM for n = 3 independent experiments, biological replicates. The statistical test was a student's t–test (unpaired, two–tailed). **(C)** Western blot analysis of the activation of the TrkB pathway in the HEK293T–CMV–TrkB cell line. Phosphorylated–ERK1/2^{MAPK} (Extracellular Signal–regulated Kinase1/2$^{Mitogen–Activated\ Protein\ Kinase}$) was detected, and Lamin A/C were used as SDS–PAGE loading controls for normalization. The band intensity (densitometry) analysis revealed a ca. 25% increase in signal intensity. Western blotting was performed for n = 2 independent experiments (biological replicate). **(D)** Effect of commercial BDNF on *Xenopus laevis* RGC axons. The data should be considered in comparison to Fig. 4E. Analysis of average axon elongation velocity as a response to PBS and commercial BDNF (100 ng/mL). n = 3 independent experiments, biological replicates. At least 47 growth cones per condition were counted. The statistical test was a two–tailed Mann–Whitney (MW), p = 0.0039. Data were not normally distributed according to the Shapiro–Wilk (SW) test; hence, the MW test was applied. Data show median with interquartile range. **(E)** Representative immunostaining microscopy images of mNS cell differentiation into neurons at 13 days *in vitro*. All scale bars indicate 50 µm. **(F)** Statistical analysis of the overall cell population for βIII–Tubulin change (images represented in panel E). Cultures treated with commercial BDNF (Com. BDNF) and PBS were considered as 100% and 0%, respectively. **(G)** Statistical analysis of the overall cell population for the change in cleaved Caspase–3 (images represented in panel E). Cultures treated with commercial BDNF and PBS were considered as 0% and 100%, respectively. All experiments were performed as independent biological replicates, n = 3. Data show mean, and error bars represent ± SEM. Statistical tests were student's t–test (unpaired, two–tailed).

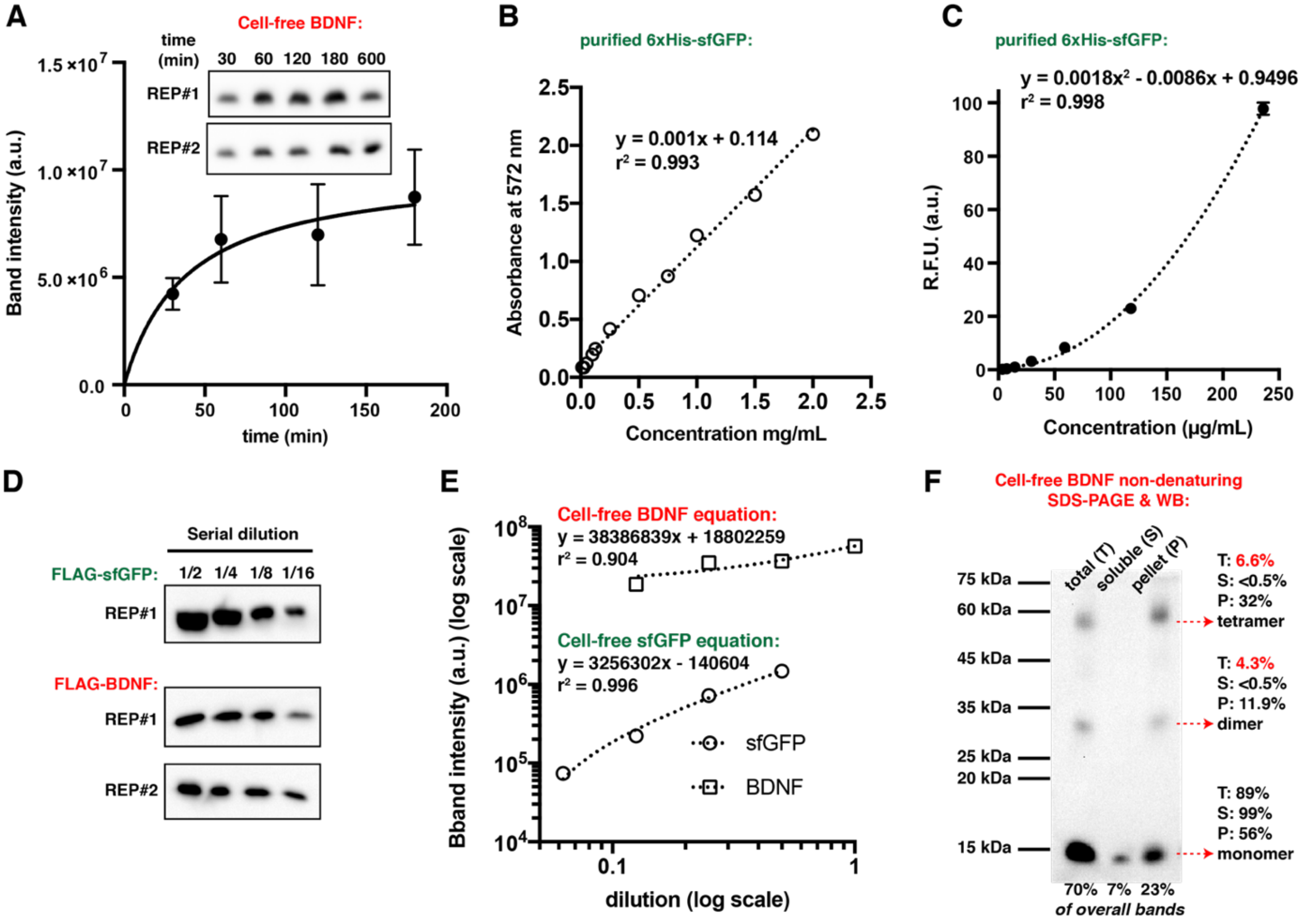

fig. S16. Quantification of cell–free synthesized BDNF. 6xHis–sfGFP was recombinantly expressed in *E. coli* BL21 (DE3) and purified with a Ni^{2+}–NTA column. Known amounts of 6xHis–sfGFP, determined by the extinction coefficient, were used to generate stock solutions. The western blotting of FLAG–tagged BDNF and FLAG–tagged sfGFP was performed with serial dilutions (1:1 dilutions) to generate a standard curve. The linear fits of blot quantification were used to estimate the amount of BDNF and sfGFP produced from the *in vitro* transcription–translation reaction. The data were then compared to known amounts of purified 6xHis–tagged sfGFP. **(A)** Time–based production of BDNF synthesized by *in vitro* transcription–translation. **(B)** Standard curve of sfGFP concentration determined by BCA analysis. **(C)** Standard curve of sfGFP fluorescence vs. protein concentration in which the fluorescence was measured with a Rotor–Gene Q qPCR machine (Qiagen). **(D)** sfGFP and BDNF western blotting with linear dilutions. Representative images or replicates are shown. **(E)** Band intensity quantification of blots from (D). Under these conditions, the concentration of cell–free expressed BDNF was ca. 100 ± 20 μg/mL. **(F)** Non–denaturing SDS–PAGE western blots for the folding of BDNF and the quantification of band intensity. Cell–free expressed BDNF corresponded to <10% of the total multimeric or soluble fraction of the protein.

Table S1. Recommended and optimized *E. coli* extract reaction conditions*.

Component	Recommended amount (*20*)	Optimized amount (this study)
Na^+–HEPES at pH 8.0	50 mM	33 mM
3–phosphoglyceric acid	30 mM	20 mM
ATP and GTP	1.5 mM each	1 mM each
CTP and UTP	0.9 mM each	0.6 mM each
E. coli MRE 600 tRNA mix	0.2 mg/mL	0.133 mg/mL
Coenzyme A	0.255 mM	0.17 mM
NAD^+	0.33 mM	0.22 mM
Cyclic AMP	0.75 mM	0.5 mM
Folinic Acid	0.675 mM	0.045 mM
Spermidine	1 mM	0.66 mM
Amino acids (each)	1.5 mM	0.5 mM
Sucrose	0 mM	20 mM
Maltose	12 mM	6 mM
PEG 8000	2% w/v	2% w/v
Mg^{2+}–glutamate	5–20 mM	10 mM
K^+–glutamate	5–20 mM	10 mM
DTT	0–4 mM	0 mM
E. coli S12 crude extract	33% v/v	33% v/v

*The components of the energy regeneration solution are in **blue**, amino acid mixture is in **red**, the supplemental components are in **green**.

Table S2. List of genetic constructs and sequences used in this work.*

DT033A	pr.Tac–m.BDNF–B0015	GTTGACAATTAATCATCGGCTCGTATAATGTGTGGgatactag*AAGAAGGAG*aataatct**ATG**GACTACAAAGACGATGATGACAAGATGCACTCCGACCCTGCCCGCCGTGGGGAGCTGAGCGTGTGTGACAGTATTAGCGAGTGGGTCACAGCGGCAGATAAAAAGACTGCAGTGGACATGTCTGGCGGGACGGTCACAGTCCTAGAGAAAGTCCCGGTATCCAAAGGCCAACTGAAGCAGTATTTCTACGAGACCAAGTGTAATCCCATGGGTTACACCAAGGAAGGCTGCAGGGGCATAGACAAAAGGCACTGGAACTCGCAATGCCGAACTACCCAATCGTATGTTCGGGCCCTTACTATGGATAGCAAAAAGAGAATTGGCTGGCGATTCATAAGGATAGACACTTCCTGTGTATGTACACTGACCATTAAGAGGGGAAGA**TAGTAG**cgactcaggctgctacgcctgtgtactggaaaacaaaaccaaaacccaaaaaacaaaaaACTGAGCCCATTGGTATCGTGGAAGGACTCgatcaaaaaaaaaaaaaaaaaaaaaaaaaaaaaaCTAGCATAACCCCTTGGGGCCTCTAAACGGGTCTTGAGGGGTTTTTTG
DT034A	pr.MW–LuxR C0062–B0015–pr.LuxR–PFO–pdt3–B0015	TTTACGGCTAGCTCAGTCCTAGGTATAGTGCTAGCgatactag*AAGAAGGAG*aataatct**ATG**GACTACAAAGACGATGATGACAAGATGAAAAACATAAATGCCGACGACACATACAGAATAATTAATAAAATTAAAGCTTGTAGAAGCAATAATGATATTAATCAATGCTTATCTGATATGACTAAAATGGTACATTGTGAATATTATTTACTCGCGATCATTTATCCTCATTCTATGGTTAAATCTGATATTTCAATCCTAGATAATTACCCTAAAAAATGGAGGCAATATTATGATGACGCTAATTTAATAAAATATGATCCTATAGTAGATTATTCTAACTCCAATCATTCACCAATTAATTGGAATATATTTGAAAACAATGCTGTAAATAAAAAATCTCCAAATGTAATTAAAGAAGCGAAAACATCAGGTCTTATCACTGGGTTTAGTTTCCCTATTCATACGGCTAACAATGGCTTCGGAATGCTTAGTTTTGCACATTCAGAAAAAGACAACTATATAGATAGTTTATTTTTACATGCGTGTATGAACATACCATTAATTGTTCCTTCTCTAGTTGATAATTATCGAAAAATAAATATAGCAAATAATAAATCAAACAACGATTTAACCAAAAGAGAAAAAGAATGTTTAGCGTGGGCATGCGAAGGAAAAAGCTCTTGGGATATTTCAAAAATATTAGGTTGCAGTGAGCGTACTGTCACTTTCCATTTAACCAATGCGCAAATGAAACTCAATACAACAAACCGCTGCCAAAGTATTTCTAAAGCAATTTTAACAGGAGCAATTGATTGCCCATACTTTAAAAAT**TAATAA**cgactcaggctgctacgcctgtgtactggaaaacaaaaccaaaacccaaaaaacaaaaaACTGAGCCCATTGGTATCGTGGAAGGACTCGATCAAAAAAAAAAAAAAAAAAAAAAAAAAAAAACTAGCATAACCCCTTGGGGCCTCTAAACGGGTCTTGAGGGGTTTTTTGgtactagaACCTGTAGGATCGTACAGGTTTACGCAAGAAAATGGTTTGTTATAGTCGAATAAAAAAtactag*AAGAAGGAG*aataatct**ATG**GACTACAAAGACGATGATGACAAGATGAAAGATATCACGGATAAAAACCAATCGATTGATAGTGGCATCAGTTCGCTGAGCTATAACCGCAATGAAGTCCTGGCCAGCAATGGTGATAAAATTGAATCGTTTGTtcctAAagaGGgtAAAAAAGCGGGCAATAAATTCATTGTAGTCGAACGCCAGAAACGTAGCCTGACTACTTCACCGGTTGATATTTCCATTATTGACAGTGTTAATGATCGCACGTATCCTGGTGCGCTGCAACTGGCTGATAAAGCATTTGTCGAAAATCGCCCTACTATTCTGATGGTAAAACGTAAACCGATCAATATCAACATTGATCTGCCGGGTCTGAAAGGTGAAAACTCTATCAAGGTGGATGATCCAACTTATGGTAAAGTCAGCGGCGCCATTGATGAACTGGTTAGCAAATGGAATGAAAAATATAGCAGCACCCACACCCTGCCGGCGCGCACCCAGTACTCCGAATCCATGGTGTATAGTAAATCCCAAATCTCATCGGCCCTGAATGTGAACGCTAAAGTGCTGGAAAATTCGCTGGGTGTTGACTTCAACGCCGTAGCAAATAACGAGAAAAAAGTAATGATCCTGGCATATAAGCAGATCTTTTATACAGTCAGTGCCGATCTGCCGAAAAATCCGTCTGATCTGTTCGATGATAGCGTTACTTTTAATGATCTGAAGCAAAAAGGTGTTAGCAACGAAGCTCCGCCGCTGATGGTTTCTAATGTTGCCTATGGTCGCACCATTTATGTTAAACTGGAAACCACTAGCTCCTCGAAAGATGTCCAAGCTGCTTTTAAAGCTCTGATCAAAAAtaCagatattAAAaaTAGTcAaCAGTATAAagaTATTTATGAAAATAGCAGTTTCACAGCAgttgtGCTGGGAGGGGATGCACAGGAACATAATAAAGTGGTGACGAAAGATTTTGACGAAATCCGCAAAGTCATCAAAGATAATGCCACTTTTagtACGAAGAATCCGGCTTATCCAATCAGCTATACCTCTGTCTTCCTGAAAGACAACAGCGTTGCCGCCGTCCATAACAAGACCGATTACATTGAAACCACCAGTACCGAATATTCTAAAGGTAAAATCAACCTGGATCATTCCGGtGCGTATGTCGCTCAGTTTGAAGTGGCGTGGGACGAGGTCTCATACGATAAAGAAGGTAACGAAGTTCTGACGCATAAAACTTGGGACGGCAATTACCAGGACAAAACTGCCCACTATAGCACCGTTATCCCGCTGGAAGCCAATGCGCGCAACATCCGCATTAAAGCACGTGAAGCAACGGGGCTGGCCTGGGAATGGTGGCGTGATGTAATTAGCGAATATGATGTGCCGCTGACGAATAATATCAACGTTTCGATTTGGGGGACCACCCTGTATCCGGGTTCAAGCATTACCTATAATGCGGCGAACAAAAACGAAGAAAACACCAACGAAGTGCCGACCTTTATGCTGAACGCGGGCCAGGCGAACAGAAGACGAGTT**TGATGA**cgactcaggctgctacgcctgtgtactggaaaacaaaaccaaaacccaaaaaacaaaaaACTGAGCCCATTGGTATCGTGGAAGGACTCgatcaaaaaaaaaaaaaaaaaaaaaaaaaaaaaaCTAGCATAACCCCTTGGGGCCTCTAAACGGGTCTTGAGGGGTTTTTTG

DT035A	pr.Tac–sfGFP–B0015	GTTGACAATTAATCATCGGCTCGTATAATGTGTGGgatactag*AAGAAGGAG*aataatct**A<br>TG**GACTACAAAGACGATGATGACAAGATGCGTAAAGGCGAAGAGCTGTTCACTGGTGTCGT<br>CCCTATTCTGGTGGAACTGGATGGTGATGTCAACGGTCATAAGTTTTCCGTGCGTGGCGAG<br>GGTGAAGGTGACGCAACTAATGGTAAACTGACGCTGAAGTTCATCTGTACTACTGGTAAAC<br>TGCCGGTACCTTGGCCGACTCTGGTAACGACGCTGACTTATGGTGTTCAGTGCTTTGCTCG<br>TTATCCGGACCATATGAAGCAGCATGACTTCTTCAAGTCCGCCATGCCGGAAGGCTATGTG<br>CAGGAACGCACGATTTCCTTTAAGGATGACGGCACGTACAAAACGCGTGCGGAAGTGAAAT<br>TTGAAGGCGATACCCTGGTAAACCGCATTGAGCTGAAAGGCATTGACTTTAAAGAAGACGG<br>CAATATCCTGGGCCATAAGCTGGAATACAATTTTAACAGCCACAATGTTTACATCACCGCC<br>GATAAACAAAAAAATGGCATTAAAGCGAATTTTAAAATTCGCCACAACGTGGAGGATGGCA<br>GCGTGCAGCTGGCTGATCACTACCAGCAAAACACTCCAATCGGTGATGGTCCTGTTCTGCT<br>GCCAGACAATCACTATCTGAGCACGCAAAGCGTTCTGTCTAAAGATCCGAACGAGAAACGC<br>GATCATATGGTTCTGCTGGAGTTCGTAACCGCAGCGGGCATCACGCATGGTATGGATGAAC<br>TGTACAAA**TAATAA**cgactcaggctgctacgcctgtgtactggaaaacaaaaccaaaaccc<br>aaaaaacaaaaaACTGAGCCCATTGGTATCGTGGAAGGACTCgatcaaaaaaaaaaaaaaa<br>aaaaaaaaaaaaaaaCTAGCATAACCCCTTGGGGCCTCTAAACGGGTCTTGAGGGGTTTTT<br>TG
DT036A	pr.Tet–sfGFP–B0015	TCCCTATCAGTGATAGAGATTGACATCCCTATCAGTGATAGAGATACTGAGCACgatacta<br>g*AAGAAGGAG*aataatct**ATG**GACTACAAAGACGATGATGACAAGATGCGTAAAGGCGAAG<br>AGCTGTTCACTGGTGTCGTCCCTATTCTGGTGGAACTGGATGGTGATGTCAACGGTCATAA<br>GTTTTCCGTGCGTGGCGAGGGTGAAGGTGACGCAACTAATGGTAAACTGACGCTGAAGTTC<br>ATCTGTACTACTGGTAAACTGCCGGTACCTTGGCCGACTCTGGTAACGACGCTGACTTATG<br>GTGTTCAGTGCTTTGCTCGTTATCCGGACCATATGAAGCAGCATGACTTCTTCAAGTCCGC<br>CATGCCGGAAGGCTATGTGCAGGAACGCACGATTTCCTTTAAGGATGACGGCACGTACAAA<br>ACGCGTGCGGAAGTGAAATTTGAAGGCGATACCCTGGTAAACCGCATTGAGCTGAAAGGCA<br>TTGACTTTAAAGAAGACGGCAATATCCTGGGCCATAAGCTGGAATACAATTTTAACAGCCA<br>CAATGTTTACATCACCGCCGATAAACAAAAAAATGGCATTAAAGCGAATTTTAAAATTCGC<br>CACAACGTGGAGGATGGCAGCGTGCAGCTGGCTGATCACTACCAGCAAAACACTCCAATCG<br>GTGATGGTCCTGTTCTGCTGCCAGACAATCACTATCTGAGCACGCAAAGCGTTCTGTCTAA<br>AGATCCGAACGAGAAACGCGATCATATGGTTCTGCTGGAGTTCGTAACCGCAGCGGGCATC<br>ACGCATGGTATGGATGAACTGTACAAA**TAATAA**cgactcaggctgctacgcctgtgtactg<br>gaaaacaaaaccaaaacccaaaaaacaaaaaACTGAGCCCATTGGTATCGTGGAAGGACTC<br>gatcaaaaaaaaaaaaaaaaaaaaaaaaaaaaaaCTAGCATAACCCCTTGGGGCCTCTAAA<br>CGGGTCTTGAGGGGTTTTTTG
DT037A	pr.ES (J23101)–sfGFP–B0015	TTTACAGCTAGCTCAGTCCTAGGTATTATGCTAGCgatactag*AAGAAGGAG*aataatct**A<br>TG**GACTACAAAGACGATGATGACAAGATGCGTAAAGGCGAAGAGCTGTTCACTGGTGTCGT<br>CCCTATTCTGGTGGAACTGGATGGTGATGTCAACGGTCATAAGTTTTCCGTGCGTGGCGAG<br>GGTGAAGGTGACGCAACTAATGGTAAACTGACGCTGAAGTTCATCTGTACTACTGGTAAAC<br>TGCCGGTACCTTGGCCGACTCTGGTAACGACGCTGACTTATGGTGTTCAGTGCTTTGCTCG<br>TTATCCGGACCATATGAAGCAGCATGACTTCTTCAAGTCCGCCATGCCGGAAGGCTATGTG<br>CAGGAACGCACGATTTCCTTTAAGGATGACGGCACGTACAAAACGCGTGCGGAAGTGAAAT<br>TTGAAGGCGATACCCTGGTAAACCGCATTGAGCTGAAAGGCATTGACTTTAAAGAAGACGG<br>CAATATCCTGGGCCATAAGCTGGAATACAATTTTAACAGCCACAATGTTTACATCACCGCC<br>GATAAACAAAAAAATGGCATTAAAGCGAATTTTAAAATTCGCCACAACGTGGAGGATGGCA<br>GCGTGCAGCTGGCTGATCACTACCAGCAAAACACTCCAATCGGTGATGGTCCTGTTCTGCT<br>GCCAGACAATCACTATCTGAGCACGCAAAGCGTTCTGTCTAAAGATCCGAACGAGAAACGC<br>GATCATATGGTTCTGCTGGAGTTCGTAACCGCAGCGGGCATCACGCATGGTATGGATGAAC<br>TGTACAAA**TAATAA**cgactcaggctgctacgcctgtgtactggaaaacaaaaccaaaaccc<br>aaaaaacaaaaaACTGAGCCCATTGGTATCGTGGAAGGACTCgatcaaaaaaaaaaaaaaa<br>aaaaaaaaaaaaaaaCTAGCATAACCCCTTGGGGCCTCTAAACGGGTCTTGAGGGGTTTTT<br>TG
DT038A	pr.EM (J23106)–sfGFP–B0015	TTTACGGCTAGCTCAGTCCTAGGTATAGTGCTAGCgatactag*AAGAAGGAG*aataatct**A<br>TG**GACTACAAAGACGATGATGACAAGATGCGTAAAGGCGAAGAGCTGTTCACTGGTGTCGT<br>CCCTATTCTGGTGGAACTGGATGGTGATGTCAACGGTCATAAGTTTTCCGTGCGTGGCGAG<br>GGTGAAGGTGACGCAACTAATGGTAAACTGACGCTGAAGTTCATCTGTACTACTGGTAAAC<br>TGCCGGTACCTTGGCCGACTCTGGTAACGACGCTGACTTATGGTGTTCAGTGCTTTGCTCG<br>TTATCCGGACCATATGAAGCAGCATGACTTCTTCAAGTCCGCCATGCCGGAAGGCTATGTG<br>CAGGAACGCACGATTTCCTTTAAGGATGACGGCACGTACAAAACGCGTGCGGAAGTGAAAT<br>TTGAAGGCGATACCCTGGTAAACCGCATTGAGCTGAAAGGCATTGACTTTAAAGAAGACGG<br>CAATATCCTGGGCCATAAGCTGGAATACAATTTTAACAGCCACAATGTTTACATCACCGCC<br>GATAAACAAAAAAATGGCATTAAAGCGAATTTTAAAATTCGCCACAACGTGGAGGATGGCA

		GCGTGCAGCTGGCTGATCACTACCAGCAAAACACTCCAATCGGTGATGGTCCTGTTCTGCT<br>GCCAGACAATCACTATCTGAGCACGCAAAGCGTTCTGTCTAAAGATCCGAACGAGAAACGC<br>GATCATATGGTTCTGCTGGAGTTCGTAACCGCAGCGGGCATCACGCATGGTATGGATGAAC<br>TGTACAAA**TAATAA**cgactcaggctgctacgcctgtgtactggaaaacaaaaccaaaaccc<br>aaaaaacaaaaaACTGAGCCCATTGGTATCGTGGAAGGACTCgatcaaaaaaaaaaaaaa<br>aaaaaaaaaaaaaaaCTAGCATAACCCCTTGGGGCCTCTAAACGGTCTTGAGGGGTTTTT<br>TG
DT039A	pr.EW (J23117)–sfGFP–B0015	TTGACAGCTAGCTCAGTCCTAGGGATTGTGCTAGCgatactag*AAGAAGGAG*aataatct**A**<br>**TG**GACTACAAAGACGATGATGACAAGATGCGTAAAGGCGAAGAGCTGTTCACTGGTGTCGT<br>CCCTATTCTGGTGGAACTGGATGGTGATGTCAACGGTCATAAGTTTTCCGTGCGTGGCGAG<br>GGTGAAGGTGACGCAACTAATGGTAAACTGACGCTGAAGTTCATCTGTACTACTGGTAAAC<br>TGCCGGTACCTTGGCCGACTCTGGTAACGACGCTGACTTATGGTGTTCAGTGCTTTGCTCG<br>TTATCCGGACCATATGAAGCAGCATGACTTCTTCAAGTCCGCCATGCCGGAAGGCTATGTG<br>CAGGAACGCACGATTTCCTTTAAGGATGACGGCACGTACAAAACGCGTGCGGAAGTGAAAT<br>TTGAAGGCGATACCCTGGTAAACCGCATTGAGCTGAAAGGCATTGACTTTAAAGAAGACGG<br>CAATATCCTGGGCCATAAGCTGGAATACAATTTTAACAGCCACAATGTTTACATCACCGCC<br>GATAAACAAAAAAATGGCATTAAAGCGAATTTTAAAATTCGCCACAACGTGGAGGATGGCA<br>GCGTGCAGCTGGCTGATCACTACCAGCAAAACACTCCAATCGGTGATGGTCCTGTTCTGCT<br>GCCAGACAATCACTATCTGAGCACGCAAAGCGTTCTGTCTAAAGATCCGAACGAGAAACGC<br>GATCATATGGTTCTGCTGGAGTTCGTAACCGCAGCGGGCATCACGCATGGTATGGATGAAC<br>TGTACAAA**TAATAA**cgactcaggctgctacgcctgtgtactggaaaacaaaaccaaaaccc<br>aaaaaacaaaaaACTGAGCCCATTGGTATCGTGGAAGGACTCgatcaaaaaaaaaaaaaa<br>aaaaaaaaaaaaaaaCTAGCATAACCCCTTGGGGCCTCTAAACGGTCTTGAGGGGTTTTT<br>TG
DT040A	pr.MW–sfGFP–B0015	TTTACGGCTAGCTCAGTCCTAGGGATTGTGCTAAGCgatactag*AAGAAGGAG*aataatct<br>**ATG**GACTACAAAGACGATGATGACAAGATGCGTAAAGGCGAAGAGCTGTTCACTGGTGTCG<br>TCCCTATTCTGGTGGAACTGGATGGTGATGTCAACGGTCATAAGTTTTCCGTGCGTGGCGA<br>GGGTGAAGGTGACGCAACTAATGGTAAACTGACGCTGAAGTTCATCTGTACTACTGGTAAA<br>CTGCCGGTACCTTGGCCGACTCTGGTAACGACGCTGACTTATGGTGTTCAGTGCTTTGCTC<br>GTTATCCGGACCATATGAAGCAGCATGACTTCTTCAAGTCCGCCATGCCGGAAGGCTATGT<br>GCAGGAACGCACGATTTCCTTTAAGGATGACGGCACGTACAAAACGCGTGCGGAAGTGAAA<br>TTTGAAGGCGATACCCTGGTAAACCGCATTGAGCTGAAAGGCATTGACTTTAAAGAAGACG<br>GCAATATCCTGGGCCATAAGCTGGAATACAATTTTAACAGCCACAATGTTTACATCACCGC<br>CGATAAACAAAAAAATGGCATTAAAGCGAATTTTAAAATTCGCCACAACGTGGAGGATGGC<br>AGCGTGCAGCTGGCTGATCACTACCAGCAAAACACTCCAATCGGTGATGGTCCTGTTCTGC<br>TGCCAGACAATCACTATCTGAGCACGCAAAGCGTTCTGTCTAAAGATCCGAACGAGAAACG<br>CGATCATATGGTTCTGCTGGAGTTCGTAACCGCAGCGGGCATCACGCATGGTATGGATGAA<br>CTGTACAAA**TAATAA**cgactcaggctgctacgcctgtgtactggaaaacaaaaccaaaacc<br>caaaaaacaaaaaACTGAGCCCATTGGTATCGTGGAAGGACTCgatcaaaaaaaaaaaaa<br>aaaaaaaaaaaaaaaaCTAGCATAACCCCTTGGGGCCTCTAAACGGTCTTGAGGGGTTTT<br>TTG
DT041A	CMV–TrkB	CGTTGACATTGATTATTGACTAGTTATTAATAGTAATCAATTACGGGGTCATTAGTTCATA<br>GCCCATATATGGAGTTCCGCGTTACATAACTTACGGTAAATGGCCCGCCTGGCTGACCGCC<br>CAACGACCCCCGCCCATTGACGTCAATAATGACGTATGTTCCCATAGTAACGCCAATAGGG<br>ACTTTCCATTGACGTCAATGGGTGGACTATTTACGGTAAACTGCCCACTTGGCAGTACATC<br>AAGTGTATCATATGCCAAGTACGCCCCCTATTGACGTCAATGACGGTAAATGGCCCGCCTG<br>GCATTATGCCCAGTACATGACCTTATGGGACTTTCCTACTTGGCAGTACATCTACGTATTA<br>GTCATCGCTATTACCATGGTGATGCGGTTTTGGCAGTACATCAATGGGCGTGGATAGCGGT<br>TTGACTCACGGGGATTTCCAAGTCTCCACCCCATTGACGTCAATGGGAGTTTGTTTTGGCA<br>CCAAAATCAACGGGACTTTCCAAAATGTCGTAACAACTCCGCCCCATTGACGCAAATGGGC<br>GGTAGGCGTGTACGGTGGGAGGTCTATATAAGCAGAGCTCTCTGGCTAACTAGAGAACCCA<br>CTGCTTACTGGCTTATCGAAATTAATACGACTCACTATAGGGAGACCCAAGCTTGGTACC**A**<br>**TG**TCGCCCTGGCTGAAGTGGCATGGACCCGCCATGGCGCGGCTCTGGGGCTTATGCCTGCT<br>GGTCTTGGGCTTCTGGAGGGCCTCTCTCGCCTGCCCGACGTCCTGCAAATGCAGTTCCGCT<br>AGGATTTGGTGTACTGAGCCTTCTCCAGGCATCGTGGCATTCCCGAGGTTGGAACCTAACA<br>GCGTTGACCCGGAGAACATCACGGAAATTCTCATTGCAAACCAGAAAAGGCTAGAAATCAT<br>CAATGAAGATGACGTTGAAGCTTACGTGGGGCTGAGAAACCTTACAATTGTGGATTCCGGC<br>TTAAAGTTTGTGGCTTACAAAGCGTTTCTGAAAAACAGCAACCTGCGGCACATAAATTTCA<br>CACGAAACAAGCTGACGAGTTTGTCCAGGAGACATTTCCGCCACCTTGACTTGTCTGACCT<br>GATCCTGACGGGTAATCCGTTCACGTGCTCCTGCGACATCATGTGGCTCAAGACTCTCCAG<br>GAGACTAAATCCAGCCCCGACACTCAGGATTTGTACTGCCTCAATGAGAGCAGCAAGAACA

		TGCCCCTGGCGAACCTGCAGATACCCAATTGTGGTCTGCCATCTGCACGTCTGGCTGCTCC<br>TAACCTCACCGTGGAGGAAGGAAAGTCTGTGACCCTTTCCTGCAGTGTGGGGGGTGACCCA<br>CTCCCCACCTTGTACTGGGACGTTGGGAATTTGGTTTCCAAGCACATGAATGAAACAAGCC<br>ACACACAGGGCTCCTTAAGGATAACGAACATTTCATCTGATGACAGTGGAAAGCAAATCTC<br>TTGTGTGGCAGAAAACCTTGTAGGAGAAGATCAAGATTCTGTGAACCTCACTGTGCATTTT<br>GCGCCAACTATCACGTTTCTCGAGTCTCCAACCTCAGATCACCACTGGTGCATTCCATTCA<br>CTGTGAGAGGCAACCCCAAGCCTGCGCTTCAGTGGTTCTACAATGGGGCCATACTGAATGA<br>GTCCAAGTACATCTGTACTAAGATCCACGTCACCAATCACACGGAGTACCATGGCTGCCTC<br>CAGCTGGATAACCCCACTCATATGAATAACGGAGACTACACCCTGATGGCCAAGAACGAGT<br>ATGGGAAGGATGAGAGACAGATCTCCGCTCACTTCATGGGCCGGCCTGGAGTCGACTACGA<br>GACAAACCCAAATTACCCTGAAGTCCTCTATGAAGACTGGACCACGCCAACTGACATTGGG<br>GATACTACGAACAAAAGTAATGAAATCCCCTCCACGGATGTTGCTGACCAAAGCAATCGGG<br>AGCATCTCTCGGTCTATGCCGTGGTGGTGATTGCATCTGTGGTGGGATTCTGCCTGCTGGT<br>GATGTTGCTCCTGCTCAAGTTGGCGAGACATTCCAAGTTTGGCATGAAAGGCCCAGCTTCG<br>GTCATCAGCAACGACGATGACTCTGCCAGCCCCCTCCACCACATCTCCAATGGGAGTAACA<br>CTCCATCTTCTTCGGAGGGCGGTCCCGACGCTGTCATTATTGGAATGACCAAGATTCCTGT<br>TATTGAAAACCCCCAGTACTTTGGCATCACCAACAGTCAGCTCAAGCCAGACACATTTGTT<br>CAGCATATCAAGAGACACAACATCGTTCTGAAGAGGGAACTTGGGGAAGGAGCCTTCGGGA<br>AAGTTTTCCTTGCCGAGTGCTACAACCTCTGCCCAGAGCAGGATAAGATCCTGGTGGCTGT<br>GAAGACGCTGAAGGACGCCAGCGACAATGCACGCAAGGACTTTCATCGGGAAGCTGAGCTG<br>CTGACCAACCTCCAGCACGAGCACATTGTCAAGTTCTACGGTGTCTGTGTGGAGGGCGACC<br>CACTCATCATGGTCTTTGAGTACATGAAGCACGGGGACCTCAACAAGTTCCTTAGGGCACA<br>CGGGCCCGACGCAGTGCTGATGGCAGAGGGTAACCCGCCCACAGAGCTGACGCAGTCGCAG<br>ATGCTGCACATCGCTCAGCAAATCGCAGCAGGTATGGTCTACCTGGCGTCCCAACACTTTG<br>TGCACCGTGACCTGGCCACCCGGAACTGCCTGGTGGGAGAGAACCTGCTGGTGAAAATTGG<br>GGACTTTGGGATGTCCCGAGATGTGTACAGCACCGACTACTATCGGGTCGGTGGCCACACA<br>ATGTTGCCCATCCGATGGATGCCTCCAGAGAGCATCATGTACAGGAAATTCACCACCGAGA<br>GCGACGTCTGGAGCCTGGGCGTTGTGTTGTGGGAGATCTTCACCTACGGCAAGCAGCCCTG<br>GTATCAGCTATCGAACAATGAGGTGATAGAGTGCATCACCCAGGGAAGAGTCCTTCAGCGG<br>CCTCGAACGTGTCCCCAGGAGGTGTATGAGCTCATGCTTGGATGCTGGCAGCGGGAACCAC<br>ACACCCGGAAGAACATCAAGAGCATCCACACCCTCCTTCAGAACTTGGCCAAGGCATCTCC<br>CGTCTACCTGGATATCCTAGGC**TAGTAG**TCTAGAGGGCCCTATTCTATAGTGTCACCTAAA<br>TGCTAGAGCTCGCTGATCAGCCTCGACTGTGCCTTCTAGTTGCCAGCCATCTGTTGTTTGC<br>CCCTCCCCCGTGCCTTCCTTGACCCTGGAAGGTGCCACTCCCACTGTCCTTTCCTAATAAA<br>ATGAGGAAATTGCATCGCATTGTCTGAGTAGGTGTCATTCTATTCTGGGGGGTGGGGTGGG<br>GCAGGACAGCAAGGGGGAGGATTGGGAAGACAATAGCAGGCATGCTGGGGATGCGGTGGGC<br>TCTATGGCTTCTGAGGCGGAAAGAACCAGCTGGGGCTCTAGGGGGTATCCCCACGCGCCCT<br>GTAGCGGCGCATTAAGCGCGGCGGGTGTGGTGGTTACGCGCAGCGTGACCGCTACACTTGC<br>C

*From DT033A to DT040A, promoter sequences are in **blue**, spacer or polyA sequences are in lowercase, ribosome binding sites are in *italic* letters, FLAG–tag sequences are in **green**, the first and the last codons of the protein sequences are in **bold**, protein degradation tag sequences are in **cyan**, FRET–pair binding sequences (to monitor mRNA levels *in vitro*) are in **purple**, and terminator sequences are highlighted in **red**. For DT041A, the Cytomegalovirus (CMV) enhancer sequence is indicated in **green**, the CMV promoter sequence is indicated in **blue**, the first and the last codons of murine TrkB sequence are in **bold**.

Table S3. Image processing details for mNS and HEK293T cells.

Immunostain/expression	Exposure time (ms)	Histogram corrections (boundary values following background subtraction)
**Nucleus (Hoechst 33258)**	2000	100 – 6500
**βIII–Tubulin**	3000	100 – 1500
**MAP2**	5000	150 – 300
**GFAP**	5000	200 – 2000
**cleaved Caspase–3**	3000	100 – 400
**GFP**	5000	200 – 500

Captions for Movies S1 and S2

Movie S1.

Representative time–lapse movie of *Xenopus* Retinal Ganglion Cell (RGC) axons, organocultured at developmental stage 24, as a response to cell–free (S12) expressed BDNF.

Movie S2.

Representative time–lapse movie of *Xenopus* Retinal Ganglion Cell (RGC) axons, organocultured at developmental stage 24, as a response to the “No DNA Template S12” reaction.

REFERENCES

1. B. C. Buddingh, J. C. M. van Hest, Artificial cells: Synthetic compartments with life-like functionality and adaptivity. *Acc. Chem. Res.* **50**, 769–777 (2017).

2. P. Schwille, J. Spatz, K. Landfester, E. Bodenschatz, S. Herminghaus, V. Sourjik, T. J. Erb, P. Bastiaens, R. Lipowsky, A. Hyman, P. Dabrock, J.-C. Baret, T. Vidakovic-Koch, P. Bieling, R. Dimova, H. Mutschler, T. Robinson, T.-Y. D. Tang, S. Wegner, K. Sundmacher, MaxSynBio: Avenues towards creating cells from the bottom up. *Angew. Chem. Int. Ed. Engl.* **57**, 13382–13392 (2018).

3. N.-N. Deng, M. Yelleswarapu, L. Zheng, W. T. S. Huck, Microfluidic assembly of monodisperse vesosomes as artificial cell models. *J. Am. Chem. Soc.* **139**, 587–590 (2017).

4. M. J. Booth, V. R. Schild, A. D. Graham, S. N. Olof, H. Bayley, Light-activated communication in synthetic tissues. *Sci. Adv.* **2**, e1600056 (2016).

5. Y. Elani, R. V. Law, O. Ces, Vesicle-based artificial cells as chemical microreactors with spatially segregated reaction pathways. *Nat. Commun.* **5**, 5305 (2014).

6. Y. Qiao, M. Li, R. Booth, S. Mann, Predatory behaviour in synthetic protocell communities. *Nat. Chem.* **9**, 110–119 (2017).

7. R. Lentini, S. P. Santero, F. Chizzolini, D. Cecchi, J. Fontana, M. Marchioretto, C. D. Bianco, J. L. Terrell, A. C. Spencer, L. Martini, M. Forlin, M. Assfalg, M. D. Serra, W. E. Bentley, S. S. Mansy, Integrating artificial with natural cells to translate chemical messages that direct *E. coli* behaviour. *Nat. Commun.* **5**, 4012 (2014).

8. H. Niederholtmeyer, C. Chaggan, N. K. Devaraj, Communication and quorum sensing in nonliving mimics of eukaryotic cells. *Nat. Commun.* **9**, 5027 (2018).

9. A. Dupin, F. C. Simmel, Signalling and differentiation in emulsion-based multi-compartmentalized in vitro genetic circuits. *Nat. Chem.* **11**, 32–39 (2019).

10. K. P. Adamala, D. A. Martin-Alarcon, K. R. Guthrie-Honea, E. S. Boyden, Engineering genetic circuit interactions within and between synthetic minimal cells. *Nat. Chem.* **9**, 431–439 (2017).

11. D. Fletcher, Which biological systems should be engineered? *Nature* **563**, 177–179 (2018).

12. T. Abraham, M. Mao, C. Tan, Engineering approaches of smart, bio-inspired vesicles for biomedical applications. *Phys. Biol.* **15**, 061001 (2018).

13. S. Cohen-Cory, S. E. Fraser, BDNF in the development of the visual system of Xenopus. *Neuron* **12**, 747–761 (1994).

14. S. McFarlane, L. McNeill, C. E. Holt, FGF signaling and target recognition in the developing Xenopus visual system. *Neuron* **15**, 1017–1028 (1995).

15. S. McFarlane, E. Cornel, E. Amaya, C. E. Holt, Inhibition of FGF receptor activity in retinal ganglion cell axons causes errors in target recognition. *Neuron* **17**, 245–254 (1996).

16. H. Park, M.-M. Poo, Neurotrophin regulation of neural circuit development and function. *Nat. Rev. Neurosci.* **14**, 7–23 (2013).

17. R. C. Robinson, C. Radziejewsji, G. Spraggon, J. Greenwald, M. R. Kostura, L. D. Burtnick, D. I. Stuart, S. Choe, E. Y. Jones, The structures of the neurotrophin 4 homodimer and the brain-derived neurotrophic factor/neurotrophin 4 heterodimer reveal a common Trk-binding site. *Protein Sci.* **8**, 2589–2597 (1999).

18. D. M. Czajkowsky, E. M. Hotze, Z. Shao, R. K. Tweten, Vertical collapse of a cytolysin prepore moves its transmembrane β-hairpins to the membrane. *EMBO J.* **23**, 3206–3215 (2004).

19. Y. Kuruma, T. Ueda, The PURE system for the cell-free synthesis of membrane proteins. *Nat. Protoc.* **10**, 1328–1344 (2015).

20. Z. Z. Sun, C. A. Hayes, J. Shin, F. Caschera, R. M. Murray, V. Noireaux, Protocols for implementing an *Escherichia coli* based TX-TL cell-free expression system for synthetic biology. *J. Vis. Exp.* **79**, e50762 (2013).

21. O. Borkowski, M. Koch, A. Zettor, A. Pandi, A. C. Batista, P. Soudier, J.-L. Faulon, Large scale active-learning-guided exploration for in vitro protein production optimization. *Nat. Commun.* **11**, 1872 (2020).

22. L. Conti, S. M. Pollard, T. Gorba, E. Reitano, M. Toselli, G. Biella, Y. Sun, S. Sanzone, Q.-L. Ying, E. Cattaneo, A. Smith, Niche-independent symmetrical self-renewal of a mammalian tissue stem cell. *PLOS Biol.* **3**, e283 (2005).

23. D. Spiliotopoulos, D. Goffredo, L. Conti, F. Di Febo, G. Biella, M. Toselli, E. Cattaneo, An optimized experimental strategy for efficient conversion of embryonic stem (ES)-derived mouse neural stem (NS) cells into a nearly homogeneous mature neuronal population. *Neurobiol. Dis.* **34**, 320–331 (2009).

24. S. Finkbeiner, S. F. Tavazoie, A. Maloratsky, K. M. Jacobs, K. M. Harris, M. E. Greenberg, CREB: A major mediator of neuronal neurotrophin responses. *Neuron* **19**, 1031–1047 (1997).

25. Q. Shan, D. R. Storm, Optimization of a cAMP response element signal pathway reporter system. *J. Neurosci. Methods* **191**, 21–25 (2010).

26. R. Lentini, N. Y. Martín, M. Forlin, L. Belmonte, J. Fontana, M. Cornella, L. Martini, S. Tamburini, W. E. Bentley, O. Jousson, S. S. Mansy, Two-way chemical communication between artificial and natural cells. *ACS Cent. Sci.* **3**, 117–123 (2017).

27. W. T. Al-Jamal, K. Kostarelos, Liposomes: From a clinically established drug delivery system to a nanoparticle platform for theranostic nanomedicine. *Acc. Chem. Res.* **44**, 1094–1104 (2011).

28. L. Sercombe, T. Veerati, F. Moheimani, S. Y. Wu, A. K. Sood, S. Hua, Advances and challenges of liposome assisted drug delivery. *Front. Pharmacol.* 6, 286 (2015).

29. N. Krinsky, M. Kaduri, A. Zinger, J. Shainsky-Roitman, M. Goldfeder, I. Benhar, D. Hershkovitz, A. Schroeder, Synthetic cells synthesize therapeutic proteins inside tumors. *Adv. Healthc. Mater.* **7**, 1701163 (2018).

30. Y. Ding, L. E. Contreras-Llano, E. Morris, M. Mao, C. Tan, Minimizing context dependency of gene networks using artificial cells. *ACS Appl. Mater. Interfaces* **10**, 30137–30146 (2018).

31. J. M. Yano, K. Yu, G. P. Donaldson, G. G. Shastri, P. Ann, L. Ma, C. R. Nagler, R. F. Ismagilov, S. K. Mazmanian, E. Y. Hsiao, Indigenous bacteria from the gut microbiota regulate host serotonin biosynthesis. *Cell* **161**, 264–276 (2015).

32. P. Strandwitz, K. H. Kim, D. Terekhova, J. K. Liu, A. Sharma, J. Levering, D. M. Donald, D. Dietrich, T. R. Ramadhar, A. Lekbua, N. Mroue, C. Liston, E. J. Stewart, M. J. Dubin, K. Zengler, R. Knight, J. A. Gilbert, J. Clardy, K. Lewis, GABA-modulating bacteria of the human gut microbiota. *Nat. Microbiol.* **4**, 396–403 (2019).

33. S. Majumder, J. Garamella, Y.-L. Wang, M. DeNies, V. Noireaux, A. P. Liu, Cell-sized mechanosensitive and biosensing compartment programmed with DNA. *Chem. Commun.* **53**, 7349–7352 (2017).

34. J. W. Hindley, D. G. Zheleva, Y. Elani, K. Charalambous, L. M. C. Barter, P. J. Booth, C. L. Bevan, R. V. Law, O. Ces, Building a synthetic mechanosensitive signaling pathway in compartmentalized artificial cells. *Proc. Natl. Acad. Sci. U.S.A.* **116**, 16711–16716 (2019).

35. E. Merlet, L. Lipskaia, A. Marchand, L. Hadri, N. Mougenot, F. Atassi, L. Liang, S. N. Hatem, R. J. Hajjar, A.-M. Lompré, A calcium-sensitive promoter construct for gene therapy. *Gene Ther.* **20**, 248–254 (2013).

36. M. Dwidar, Y. Seike, S. Kobori, C. Whitaker, T. Matsuura, Y. Yokobayashi, Programmable artificial cells using histamine-responsive synthetic riboswitch. *J. Am. Chem. Soc.* **141**, 11103–11114 (2019).

37. C. Frantz, K. M. Stewart, V. M. Weaver, The extracellular matrix at a glance. *J. Cell Sci.* **123**, 4195–4200 (2010).

38. M. L. Jacobs, M. A. Boyd, N. P. Kamat, Diblock copolymers enhance folding of a mechanosensitive membrane protein during cell-free expression. *Proc. Natl. Acad. Sci. U.S.A.* **116**, 4031–4036 (2018).

39. P. Gobbo, A. J. Patil, M. Li, R. Harniman, W. H. Briscoe, S. Mann, Programmed assembly of synthetic protocells into thermoresponsive prototissues. *Nat. Mater.* **17**, 1145–1153 (2018).

40. G. Villar, A. D. Graham, H. Bayley, A tissue-like printed material. *Science* **340**, 48–52 (2013).

41. S. M. Garraway, J. R. Huie, Spinal plasticity and behavior: BDNF-induced neuromodulation in uninjured and injured spinal cord. *Neural Plast.* **2016**, 9857201 (2016).

42. I. Voskoboinik, J. C. Whisstock, J. A. Trapani, Perforin and granzymes: Function, dysfunction and human pathology. *Nat. Rev. Immunol.* **15**, 388–400 (2015).

43. T. Jaroentomeechai, J. C. Stark, A. Natarajan, C. J. Glasscock, L. E. Yates, K. J. Hsu, M. Mrksich, M. C. Jewett, M. P. De Lisa, Single-pot glycoprotein biosynthesis using a cell-free transcription-translation system enriched with glycosylation machinery. *Nat. Commun.* **9**, 2686 (2018).

44. J. A. Zuris, D. B. Thompson, Y. Shu, J. P. Guilinger, J. L. Bessen, J. H. Hu, M. L. Maeder, J. K. Joung, Z.-Y. Chen, D. R. Liu, Cationic lipid-mediated delivery of proteins enables efficient protein-based genome editing *in vitro* and *in vivo*. *Nat. Biotechnol.* **33**, 73–80 (2015).

45. R. Rouet, B. A. Thuma, M. D. Roy, N. G. Lintner, D. M. Rubitski, J. E. Finley, H. M. Wisniewska, R. Mendonsa, A. Hirsh, L. de Oñate, J. C. Barrón, T. J. McLellan, J. Bellenger, X. Feng, A. Varghese, B. A. Chrunyk, K. Borzilleri, K. D. Hesp, K. Zhou, N. Ma, M. Tu, R. Dullea, K. F. McClure, R. C. Wilson, S. Liras, V. Mascitti, J. A. Doudna, Receptor-mediated delivery of CRISPR-Cas9 endonuclease for cell-type-specific gene editing. *J. Am. Chem. Soc.* **140**, 6596–6603 (2018).

46. O. S. Fenton, K. N. Olafson, P. S. Pillai, M. J. Mitchell, R. Langer, Advances in biomaterials for drug delivery. *Adv. Mater.* **7**, 1705328 (2018).

47. D. G. Gibson, L. Young, R.-Y. Chuang, J. C. Venter, C. A. Hutchison III, H. O. Smith, Enzymatic assembly of DNA molecules up to several hundred kilobases. *Nat. Methods* **6**, 343–345 (2009).

48. L. A. Shepard, A. P. Heuck, B. D. Hamman, J. Rossjohn, M. W. Parker, K. R. Ryan, A. E. Johnson, R. K. Tweten, Identification of a membrane-spanning domain of the thiol-activated pore-forming toxin *Clostridium perfringens* perfringolysin O: An α-helical to β-sheet transition identified by fluorescence spectroscopy. *Biochemistry* **37**, 14563–14574 (1998).

49. T. Ellinger, R. Ehricht, Single-step purification of T7 RNA polymerase with a 6-histidine tag. *Biotechniques* **24**, 718–720 (1998).

50. M. Hadorn, E. Boenzli, K. T. Sørensen, D. De Lucrezia, M. M. Hanczyc, T. Yomo, Defined DNA-mediated assemblies of gene-expressing giant unilamellar vesicles. *Langmuir* **29**, 15309–15319 (2013).

51. A. C. Spencer, P. Torre, S. S. Mansy, The encapsulation of cell-free transcription and translation machinery in vesicles for the construction of cellular mimics. *J. Vis. Exp.* **80**, e51304 (2013).

52. T. Sunami, T. Matsuura, H. Suzuki, T. Yomo, Synthesis of functional proteins within liposomes. *Methods Mol. Biol.* **607**, 243–256 (2010).

53. S. Fujii, T. Matsuura, T. Sunami, T. Nishikawa, Y. Kazuta**,** T. Yomo, Liposome display for *in vitro* selection and evolution of membrane proteins. *Nat. Protoc.* **9**, 1578–1591 (2014).

54. Y. Matsushita-Ishiodori, M. M. Hanczyc, A. Wang, J. W. Szostak, T. Yomo, Using imaging flow cytometry to quantify and optimize giant vesicle production by water-in-oil emulsion transfer methods. *Langmuir* **35**, 2375–2382 (2019).

55. I. C. Kurt, I. Sur, E. Kaya, A. Cingoz, S. Kazancioglu, Z. Kahya, O. D. Toparlak, F. Senbabaoglu, Z. Kaya, E. Ozyerli, S. Karahüseyinoglu, N. A. Lack, Z. H. Gümüs, T. T. Onder, T. Bagci-Onder, an H3K36-specific demethylase, regulates apoptotic response of GBM cells to TRAIL. *Cell Death Dis.* **8**, e2897 (2017).

56. P. D. Nieuwkoop, J. Faber, *Normal Table of* Xenopus laevis *(Daudin): A Systematical and Chronological Survey of the Development from the Fertilized Egg Till the End of Metamorphosis* (Garland Pub, 1994).

57. A. Bellon, A. Iyer, S. Bridi, F. C. Y. Lee, C. Ovando-Vázquez, E. Corradi, S. Longhi, M. Roccuzzo, S. Strohbuecker, S. Naik, P. Sarkies, E. Miska, C. Abreu-Goodger, C. E. Holt, M.-L. Baudet, miR-182 regulates slit2-mediated axon guidance by modulating the local translation of a specific mRNA. *Cell Rep.* **18**, 1171–1186 (2017).

58. M.-L. Baudet, K. H. Zivraj, C. Abreu-Goodger, A. Muldal, J. Armisen, C. Blenkiron, L. D. Goldstein, E. A. Miska, C. E. Holt, miR-124 acts through CoREST to control onset of Sema3A sensitivity in navigating retinal growth cones. *Nat. Neurosci.* **15**, 29–38 (2012).

59. D. S. Campbell, C. E. Holt, Chemotropic responses of retinal growth cones mediated by rapid local protein synthesis and degradation. *Neuron* **32**, 1013–1026 (2001).

60. Ö. D. Toparlak, M. Karki, V. E. Ortuno, R. Krishnamurthy, S. S. Mansy, Cyclophospholipids increase protocellular stability to metal ions. *Small* **16**, 1903381 (2020).

61. T. Mosmann, Rapid colorimetric assay for cellular growth and survival: Application to proliferation and cytotoxicity assays. *J. Immunol. Methods* **65**, 55–63 (1983).

62. D. E. Cameron, J. J. Collins, Tunable protein degradation in bacteria. *Nat. Biotechnol.* **32**, 1276–1281 (2014).

63. E. Altamura, P. Carrara, F. D'Angelo, F. Mavelli, P. Stano, Extrinsic stochastic factors (solute partition) in gene expression inside lipid vesicles and lipid-stabilized water-in-oil emulsion droplets: A review. *Synth. Biol.* **3**, ysy011 (2018).
